# Statistical Mechanics and Thermodynamics: Boltzmann’s versus Planck’s State Definitions and Counting †

**DOI:** 10.3390/e23070875

**Published:** 2021-07-08

**Authors:** Peter Enders

**Affiliations:** Institute of Physics, Mathematics and Informatics, Kazakh National Pedagogical Abai University, Almaty 050010, Kazakhstan; enders.kbd@gmx.de

**Keywords:** Bose–Einstein statistics, complexion, configuration number, indistinguishability, interchangeability, microstate, Maxwell–Boltzmann statistics, occupancy number, occupation number, Planck distribution

## Abstract

During the physical foundation of his radiation formula in his December 1900 talk and subsequent 1901 article, Planck refers to Boltzmann’s 1877 combinatorial-probabilistic treatment and obtains his *quantum* distribution function, while Boltzmann did not. For this, Boltzmann’s memoirs are usually ascribed to classical statistical mechanics. Agreeing with Bach, it is shown that Boltzmann’s 1868 and 1877 calculations can lead to a Planckian distribution function, where those of 1868 are even closer to Planck than that of 1877. Boltzmann’s and Planck’s calculations are compared based on Bach’s three-level scheme ‘configuration–occupation–occupancy’. Special attention is paid to the concepts of interchangeability and the indistinguishability of particles and states. In contrast to Bach, the level of exposition is most elementary. I hope to make Boltzmann’s work better known in English and to remove misunderstandings in the literature.

## 1. Introduction

In the relationship between statistical mechanics and thermodynamics, Ludwig Boltzmann’s and Max Planck’s works play a leading role. In his combinatorial calculations for founding his radiation law, Planck [1] refers to Boltzmann’s 1877 [2] definition and counting of states and the definition of entropy based thereon. Planck obtained his quantum distribution law, while Boltzmann did not.

Perhaps for that reason, Boltzmann’s work is usually connected with the classical Maxwell–Boltzmann statistics, rather than the quantum Bose–Einstein statistics, an exception to this being Bach [3] (see also [4] (chp. 5.1.2)). In agreement with Bach but at an elementary level, I will show that Boltzmann’s 1868 [5] and 1877 [2] definitions and counting of states can lead to a Planckian distribution law, where the 1868 memoir [5] is even much closer to Planck’s 1901 treatment [6] than the 1877 memoir [2]. In addition, while there is an English translation of the seminal 1877 memoir [7], I am not aware of an English translation of the pioneering 1868 memoir [5] that marks the beginning of Boltzmann’s application of combinatorics in statistical mechanics.

It is thus worthy to compare Boltzmann’s and Planck’s manners of state definition and counting. I will exploit the following three levels of description [3] (see also [4] (chp. 3.2)): configuration–occupation–occupancy.

I hope that this will elucidate Boltzmann’s papers, which are often difficult to read. For instance, Boltzmann 1877 [2] switches without notification between configuration, occupation, and occupancy, which has led to misinterpretations in the literature. 

“Boltzmann’s qualities as an outstanding lecturer are not reflected in his scientific papers, which are sometimes unduly long, occasionally obscure, and often dense. Their main conclusions are sometimes tucked away among lengthy calculations.” [8] (p. 65). “The [1877] paper … is a prime example of this description.” [9] (p. 4). “By the study of Boltzmann I have been unable to understand him. He could not understand me on account of my shortness, and his length was and is an equal stumbling block to me.”^1^

To be fair, there are exceptions. “I might add that these two pages [p. 84f] in Boltzmann’s memoir of 1868 rebut criticisms of Boltzmann’s verbosity; they show just how condensed Boltzmann’s style could be.” [3] (p. 15).

The level of explanation is elementary; readers with advanced mathematical knowledge of statistics should also consult Bach’s 1997 monograph “Indistinguishable Classical Particles” [4] where there is much more in-depth material.

Boltzmann’s and Planck’s state descriptions that are considered here were based on combinatorics.^2^ The basic sets are the multiplets.

“Multiplets. Given $n_{1}$ elements $a_{1},\cdots ,a_{n_{1}}$ and $n_{2}$ elements $b_{1},\cdots ,b_{n_{2}}$, etc., up to $n_{r}$ elements $x_{1},\cdots ,x_{n_{r}}$, it is possible to form $n_{1}\cdot n_{2}\cdots n_{r}$ ordered *r*-tuplets $\left(a_{j_{1}},b_{j_{2}}\cdots ,x_{j_{r}}\right)$ containing one element of each kind.” [13] (p. 27, II. 1).

In other words, “*r* successive selections (decisions) with exactly $n_{k}$ choices possible at the *k*th step can produce a total of $n_{1}\cdot n_{2}\cdots n_{r}$ different results.”

(1) $$Q_{0}(n_{1},n_{2},\cdots ,n_{r}):=n_{1}\cdot n_{2}\cdots n_{r}$$

Some of the formulae below will be derived from this fundamental result.

## 2. The Three Description Levels: Configuration–Occupation–Occupancy

According to Bach [4] (chp. 3.2.1 and 5.1; see also [3]), Boltzmann 1877^3^ has invented the scheme (*λ*, *n*) for the problem of *λ* equal particles being distributed onto *n* equal cells.^4^

This scheme involves the following three levels of descriptions:

1. **Configuration**: *which* particle is in *which* cell? Both the particles and the cells are distinguished and thus non-interchangeable.
2. **Occupation**: *how many* particles are in *which* cell? The cells are still distinguished, while the particles are not distinguished and thus interchangeable (used by Boltzmann [2,5] and Planck [1,6]).
3. **Occupancy**: *how many* cells host *how many* (0, 1, 2, …) particles? The particles and the cells containing the same number of particles are not distinguished and thus interchangeable (used by Boltzmann 1877 [2]).

Thus, the wording “*which*” refers to distinguished, non-interchangeable particles and cells, respectively, while “how many” refers to not distinguished, interchangeable particles.

### 2.1. Level 1: Configuration

A configuration describes which particle is in which cell. It represents a complete description of the distribution of the particles into the cells. It is supposed that the cells and the particles are distinguished (e.g., through numbers), i.e., they are not interchangeable.

This description can be realized through an λ×n matrix, **M**, where $M_{ir}=1\left(0\right)$, if particle *i* is (is not) in cell *r* (i=1,2,⋯,λ, r=1,2,⋯,n). This matrix can be condensed into the configuration number vector, $j=\left(j_{1},j_{2},\cdots ,j_{\lambda }\right)$, where $j_{i}$ is the number of the cell, in which particle *i* is located.

(2) $$j_{i}=\sum_{r=1}^{n}M_{ir}r;i=1\ldots \lambda ;1\leq j_{i}\leq n$$

Each of the *λ* configuration numbers, $j_{i}$, can assume *n* different values. For this, there are altogether the following number of different configurations:

(3) $$Q_{1}(\lambda |n):=Q_{0}(\underset{\begin{array}{cc} \lambda & \mathrm{times} \end{array}}{\underbrace{n,n,\cdots ,n}})=n^{\lambda }$$

where *Q*_0_ is defined in Formula (1). In contrast to the configuration number of random variables [4] (p. 58), the particles in a configuration are distinguished. For the sake of simplicity, as well as for the goal of this contribution, it is not necessary to introduce the random variables.

All of the configurations are considered to have equal a priori probabilities. As a consequence, the probability of the occurrence of a given configuration, **j**, is independent of **j**.

(4) $$P_{\mathrm{MB}}(j)=\frac{1}{Q_{1}(\lambda |n)}=\frac{1}{n^{\lambda }}$$

The index “MB” indicates that this defines Maxwell–Boltzmann statistics (cf. [4], p. 2, formula (2)).

By virtue of the independence of $P_{MB}\left(j\right)$ from **j**, one obtains the following result, which, given the common textbook representations, is surprising given solely this probability: the particles of the Maxwell–Boltzmann statistics *are* interchangeable, although they are *not* interchangeable in the configurations.

There are limits for the configuration numbers, see their definition in (2), but, in contrast to the occupation and occupancy numbers below, there are no constraints.

**Scheme 1:**
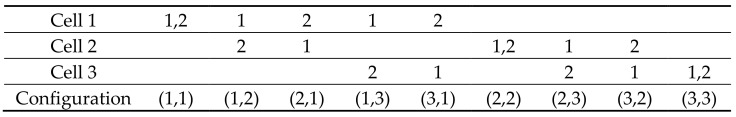
The nine different distributions of two distinguished, non-interchangeable particles onto three distinguished, non-interchangeable cells (configurations).

The $3^{2}=9$ configurations for two distinguished, non-interchangeable particles (numbered as 1 and 2) in two distinguished, non-interchangeable cells (numbered as 1, 2, and 3) are listed in Scheme 1.

### 2.2. Level 2: Occupation

**Scheme 2:**
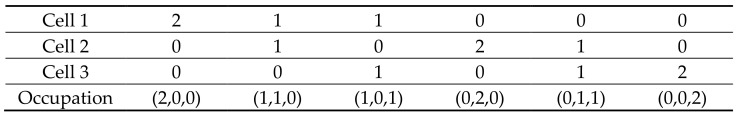
The six different distributions of two *not* distinguished, interchangeable particles onto three distinguished, non-interchangeable cells (occupations).

Often, the particles are *not* distinguished, i.e., interchangeable (they may be numbered or not). For instance, the red balls in a snooker game are distinguishable by their positions on the table. Nevertheless, an interchange of two of them does not influence the outcome of the game; they are interchangeable by definition. In such a case, the configurations (1,2) and (2,1), (1,3) and (3,1), and (2,3) and (3,2) in Scheme 1 become equivalent. This reduces the number of different distributions to six (see Scheme 2).

In contrast, the cells are still distinguished, but not interchangeable. That means that the occupations of (2,0,0), (0,2,0) and (0,0,2) are considered to be different.

It can only be said, *how many* particles are in each of the three cells: zero, one, or two particles. This information is recorded in the occupation number vector, $k=\left(k_{1},k_{2},\cdots ,k_{n}\right)$. There are $k_{r}$ particles in cell *r*.

If a configuration, **M** or **j**, is given, the corresponding occupation numbers are derived as follows:

(5) $$k_{r}(j)=\sum_{i=1}^{\lambda }M_{ir}=\sum_{i=1}^{\lambda }\delta_{j_{i},r};r=1\ldots n;0\leq k_{r}(j)\leq \lambda$$

For two particles in three cells, the occupation number vectors are given in row four of Scheme 2 above.

As the total number of particles is given to equal *λ*, there is the obvious constraint

(6) $$\sum_{r=1}^{n}k_{r}=\lambda$$

**Scheme 3:**
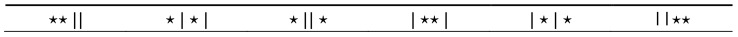
Representation of the six occupation number vectors in Scheme 2 as a sequence of stars and bars. The number of stars to the left of the first bar is equal to the number of particles in cell 1. The number of stars to the right of the first bar and to the left of the second bar is equal to the number of particles in cell 2. The number of stars to the right of the second bar is equal to the number of particles in cell 3.

Now, the occupation number vectors correspond to *n*-tuples of non-negative integers, whose sum is *λ*. The number of such *n*-tuples is equal to a multiset number.

(7) $$J=Q_{2}(\lambda |n):=\left(\left(\begin{array}{c} \lambda +1\\ n-1 \end{array}\right)\right):=\left(\begin{array}{c} \lambda +n-1\\ n-1 \end{array}\right)=\frac{\left(\lambda +n-1\right)!}{\lambda !\left(n-1\right)!}=\left(\begin{array}{c} n+\lambda -1\\ \lambda \end{array}\right)=\left(\left(\begin{array}{c} n\\ \lambda \end{array}\right)\right)=Q_{2}(n-1|\lambda +1),$$

where *J* is Boltzmann’s 1877 *J* [2] (p. 1983). This formula is easily proven by the method of stars and bars.^5^ In Scheme 2 above, one represents the two particles by two stars in a line and the three cells by two separating bars between the stars. The six cases in Scheme 2 are depicted by the six figures in Scheme 3.

One does not need *n*, but only *n*-1 bars to symbolize the *n* cells. For this, the figures have λ+n−1 characters (stars and bars). The n−1 bars can be put on λ+1 places. This is equivalent of drawing n−1 multisets from a set of size λ+1. In other words, “the number of distinguishable distributions equals the number of ways of selecting *r* [*λ*] places out of n+r−1 [n+λ−1].” [13] (p. 38).

If all of the occupations are considered to be equally likely (the occupation numbers being uniformly distributed), the probability for a given occupation, **k**, to occur is the same for all of the occupations and, hence, are independent of **k**.

(8) $$P_{\mathrm{BE}}(k)=\frac{1}{Q_{2}(\lambda |n)}={\left(\begin{array}{c} n+\lambda -1\\ \lambda \end{array}\right)}^{-1}$$

The index “BE” indicates that this probability is a definition of Bose–Einstein statistics [4] (p. 3). This is the probabilistic scheme that Planck 1901 [6] used. We will see in Section 3 that Boltzmann in 1868 [5] was quite close to that.

The algebraic symmetry of $Q_{2}\left(\lambda |n\right)=Q_{2}\left(n-1|\lambda +1\right)$ suggests that $Q_{2}\left(\lambda |n\right)$ also applies to the distribution of n−1 particles onto λ+1 cells, and, moreover, to the distribution of n−1 cells onto λ+1 particles. However, constraint (6) would have to be changed accordingly. In the second case, one deals not with interchangeable particles in distinguished cells, but with distinguished particles in interchangeable cells. This can be arranged in urn games but is physically hard to explain when the particles are Planck’s energy elements, and the cells are resonators. I will return to this ambiguity when discussing Boltzmann’s and Planck’s combinatorics.

The absence of configurations in the definition (8) of Bose–Einstein statistics does not mean that their presence is characteristic of Maxwell–Boltzmann statistics. One can include configurations through postulating the permutation invariant distribution (cf. [4], formula (1.6))

(9) $$P_{\lambda }(j):={\left(\begin{array}{c} \lambda \\ k_{1}(j)\ldots k_{n}(j) \end{array}\right)}^{-1}{\left(\begin{array}{c} n+\lambda -1\\ \lambda \end{array}\right)}^{-1}$$

The first factor is the inverse of the number of ways in which the *λ* particles can be distributed on *n* ordered parts, of which the *r*th part contains $k_{r}$ particles.

(10) $$P_{\lambda }(k):=\left(\begin{array}{c} \lambda \\ k_{1}(j)\ldots k_{n}(j) \end{array}\right):=\frac{\lambda !}{k_{1}!k_{2}!\cdots k_{n}!};k=(k_{1},k_{2},\cdots ,k_{n})$$

In this multinomial coefficient, *λ*! is the number of permutations of *λ* distinguished, non-interchangeable particles. The denominator indicates that the particles in the parts are interchangeable so that the permutations among them do not yield new distributions.

There are $P_{\lambda }\left(k\right)$ configurations for each given occupation number vector, **k** [4] (p. 59).

Feller ([13], p. 37, II. 4) gives the following simple proof for formula (10). There are $Q_{1}\left(k_{1}|\lambda \right)$ possible parts of size $k_{1}$ in the set of *λ* particles, $Q_{1}\left(k_{2}|\lambda -k_{1}\right)$ possible parts of size *k*_2_ in the remaining set of $\lambda -k_{1}$ particles, etc., until there are $Q_{1}\left(k_{n-1}|\lambda -k_{1}-k_{2}-\cdots -k_{n-2}\right)$ possible parts of size *k_n_*_−1_ in the remaining set of $\lambda -k_{1}-k_{2}-\cdots -k_{n-2}$ particles. The last $k_{n}$ particles form the last, the *n*th, part with the following possibility:

(11) $$Q_{1}\left(k_{n}|\lambda -k_{1}-\ldots -k_{n}{}_{-1}\right)=\frac{\left(\lambda -k_{1}-\ldots -k_{n}{}_{-1}\right)!}{\left(\lambda -k_{1}-\ldots -k_{n}{}_{-1}-k_{n}\right)!k_{n}!}=\frac{k_{n}!}{0!k_{n}!}=1$$

This amounts to the following number of parts:

(12) $$\begin{array}{cc} \; & P_{\lambda }\left(k\right)=\underset{Q_{1}\left(k_{1}|\lambda \right)}{\underset{︸}{\frac{\lambda !}{\left(\lambda -k_{1}\right)!k_{1}!}}}\cdot \underset{Q_{1}\left(k_{2}|\lambda -k_{1}\right)}{\underset{︸}{\frac{\left(\lambda -k_{1}\right)!}{\left(\lambda -k_{1}-k_{2}\right)!k_{2}!}}}\cdot \underset{Q_{1}\left(k_{3}|\lambda -k_{1}-k_{2}\right)}{\underset{︸}{\frac{\left(\lambda -k_{1}-k_{2}\right)!}{\left(n-k_{1}-k_{2}-k_{3}\right)!k_{3}!}}}\cdot \ldots \\ \; & \cdot \underset{Q_{1}\left(k_{n-1}|\lambda -k_{1}-k_{2}-k_{3}-\ldots -k_{n-2}\right)}{\underset{︸}{\frac{\left(\lambda -k_{1}-k_{2}-k_{3}-\ldots -k_{n-2}\right)!}{\left(\lambda -k_{1}-k_{2}-k_{3}-\ldots -k_{n-2}-{k_{n}}_{-1}\right)!{k_{d}}_{-1}!}}}\cdot \underset{Q_{1}\left(k_{n}|\lambda -k_{1}-k_{2}-k_{3}-\ldots -k_{n-1}\right)}{\underset{︸}{\frac{\left(\lambda -k_{1}-k_{2}-k_{3}-\ldots -k_{n-1}\right)!}{\left(\lambda -k_{1}-k_{2}-k_{3}-\ldots -k_{n-1}-k_{n}\right)!k_{n}!}}} \end{array}$$

I have added the last factor, $Q_{1}\left(k_{n-1}|\lambda -k_{1}-k_{2}-\cdots -k_{n-2}\right)=1$ (11), in order to make obvious the mutual cancellation of the factor $\left(\lambda -k_{1}-k_{2}-\cdots -k_{r}\right)!$ in the numerator of the *r*th term and the denominator of the (*r*+1)th term $\left(r=1,2,\cdots ,n-1\right)$.

Since $P_{\lambda }\left(k\right)$ (10) depends on **k**, the occupations, **k**, have different likelihoods, as the occupation numbers are being *not* uniformly distributed. Correspondingly, the equipartition law applies to classical, but not to quantum statistical mechanics (cf. the discussion of Boltzmann’s 1868 memoir [5] in chp. 3).

### 2.3. Level 3: Occupancy

**Scheme 4:**
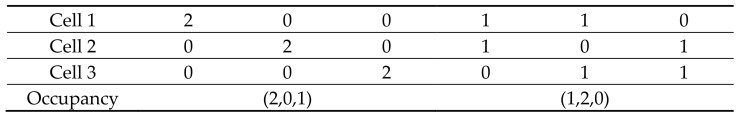
The two different distributions of two *not* distinguished, interchangeable particles onto three cells which are distinguished solely by the number of particles in them (occupancies). Left: two cells host no particles, zero cells host one particle, one cell hosts two particles. Right: one cell hosts no particles, two cells host one particle, zero cells host two particles.

The occupation numbers still treat the cells as being distinguished (e.g., by the cell numbers in Scheme 2), i.e., not interchangeable. In certain situations, however, it is not necessary or desirable to distinguish them. Then, the cells differ solely by the number of particles hosted, and all of the cells with the same number of particles are no longer distinguished, i.e., interchangeable. The question then is, *how many* cells host zero particles, one particle... *λ* particles? This question is answered by the occupancy number vector, $w=\;\left(w_{0},w_{1},\cdots ,w_{\lambda }\right)$ (see Scheme 4).

If occupations, **k**, are defined, there are $w_{s}$ cells with *s* particles, where ([4], formula (3.55))

(13) $$w_{s}(k)=\sum_{r=1}^{n}\delta_{k_{r},s};s=0,1,\ldots \lambda ;0\leq w_{s}\leq n.$$

The reduction of information from configurations to occupations has led to constraint (6). The further reduction of information from occupation to occupancy results in the presence of *two* constraints.

(14) $$\sum_{s=0}^{\lambda }w_{s}=n\begin{array}{ccc} & \mathrm{and} & \end{array}\sum_{s=0}^{\lambda }sw_{s}=\lambda$$

where $w_{s}/n$ is the probability that a molecule has the energy sε.

For each given occupancy number vector, **w**, there is the following amount of different occupation number vectors, **k** ([2], formula (3)):

(15) $$Q_{3}(w|n):=P_{n}(w):=\left(\begin{array}{c} n\\ w_{0}\ldots w_{\lambda } \end{array}\right)=\frac{n!}{w_{0}!w_{1}!\cdots w_{\lambda }!}$$

For there are *n*! permutations of the *n* cells. All of the cells with the same number of particles hosted are interchangeable, so that their permutation does not yield a different distribution. 

This “thermodynamic probability” (Planck) (15) depends on **w**, i.e., the occupancy number vectors are not uniformly distributed. 

We are now prepared to compare Boltzmann’s [2,5] definitions with Planck’s [1,6] treatments, in particular, their basic entity, the “complexion”, on an equal footing. 

## 3. Boltzmann’s 1868 State Definition and Counting

Before analyzing Boltzmann’s 1877 [2] state definitions and counting, it is most useful to consider his 1868 [5] approach.

As a matter of fact, this memoir concentrates on the distribution on phase space and describes “what is now called the microcanonical distribution in the 6n−1 energy surface” [16] (p. 4; see also [10,17]). I will omit all parts of the text that are not related to discrete state definition and counting. I am not aware of any English translations of this pioneering memoir. For this, I will refer to the original text in [18] (vol. I). For the sake of an easier comparison to the original text, I will retain Boltzmann’s symbols.

In Section II.1, Boltzmann considers a *finite* number, *n*, of “material points”, also known as particles. The kinetic energy of particle *r* is $k_{r}$, r=1,2,⋯,n (he writes “living force” but equals that to $mv^{2}/2$). The total kinetic energy of all particles is *nκ*, where *κ* is the average kinetic energy of a single particle. This implies the following constraint (p. 83; (B-*n*) is the *n*th display formula in Section II or is very similar to it):

(16) $$k_{1}+k_{2}+k_{3}+\ldots +k_{n}=n\kappa ;0\leq k_{r}\leq n\kappa \;(B-13)$$

Then, he divides the total kinetic energy, *nκ*, into “infinitely many (*p*) equal parts” (p. 84), ε=nκ/p. In terms of them, constraint (16) reads as follows:

(17) $$\frac{k_{1}}{\varepsilon }+\frac{k_{2}}{\varepsilon }+\frac{k_{3}}{\varepsilon }+\ldots +\frac{k_{n}}{\varepsilon }=p;0\leq \frac{k_{r}}{\varepsilon }\leq p$$

This is an analogue to constraint (6). The occupation number, $k_{r}/\varepsilon$, is the number of energy parts, *ε*, on particle *r*. Bach [3] (pp. 9, 18) writes that Boltzmann works with occupancy numbers. However, there is no second constraint similar to (14). The combinatorial scheme is this:

$$n\;\mathrm{particles}\;\mathrm{in}\;d\;\mathrm{cells}\;\hat{=}p\mathrm{energy}\mathrm{parts}\mathrm{on}n\mathrm{molecules}$$

In contrast to Planck 1900 [1], no physical meaning is given to the energy portions, ε=nκ/p. For this, this partition is a discretization rather than a quantization, and the continuum limit, ε→0, is possible (and will eventually be performed by Boltzmann).

At this time, combinatorics was not yet systematically developed; therefore, Boltzmann himself had to find the formulas for his problem. He analyzed the possible distributions for two, three, and four particles and generalized the formulae obtained to an arbitrary number of particles. For the sake of completeness and to ease the generalization, I add the trivial problem for one particle.

### 3.1. 1 Particle

For n=1, condition (16) implies that $k_{1}=\kappa$. This is the only possibility; thus, the number of different distributions is

(18) $$1=Q_{2}(0|p)=Q_{2}(p-1|1),$$

where $Q_{2}$ is defined in Formula (7). I will exploit the algebraic symmetry of $Q_{2}$ to explain why Boltzmann’s final result deviates from the general result (7) later on.

### 3.2. 2 Particles

For n=2, $k_{1}$ lies in one of the following intervals, supposedly with equal probability for each interval:

(19) $$\left(0\ldots 1\right)\frac{2\kappa }{p},\left(1\ldots 2\right)\frac{2\kappa }{p},\left(2\ldots 3\right)\frac{2\kappa }{p},etc.$$

Since $k_{2}$ is fixed by constraint (16), $k_{2}=2\kappa -k_{1}$, it does not involve any further possibilities. There are *p* intervals and hence there is the following amount of different distributions:

(20) $$p=Q_{2}(1|p)=Q_{2}(p-1|2)$$

### 3.3. 3 Particles

For *n* = 3, there are the following three cases:

**(Case 1)**$k_{1}$ lies in the uppermost, first from above, energy interval:

(21) $$\left(p-1\right)\frac{3\kappa }{p}\leq k_{1}\leq p\frac{3\kappa }{p}=3\kappa$$

By the constraint (16), $k_{2}$ lies in the lowest energy interval:

(22) $$0\frac{3\kappa }{p}=0\leq k_{2}\leq 1\frac{3\kappa }{p}=\frac{3\kappa }{p}$$

This represents one possibility for $k_{2}$; $k_{3}$ being always determined by the constraint (16).

**(Case 2)**$k_{1}$ lies in the second-highest energy interval:

(23) $$\left(p-2\right)\frac{3\kappa }{p}\leq k_{1}\leq \left(p-1\right)\frac{3\kappa }{p}\;(B-19)$$

By virtue of the constraint (16), $k_{2}$ may lie in the lowest, *or* in the second-lowest energy intervals. This represents two possibilities for $k_{2}$.

**(Case 3)**$k_{1}$ lies in the third-highest energy interval. By virtue of the constraint (16), again, there are three different possibilities for $k_{2}$, and so on for all of the *p* energy intervals.

All of these cases are supposed to have the same probability. For this, there is, altogether, the following amount of distributions:

(24) $$1+2+3+\ldots +p=\frac{p\left(p+1\right)}{2}=Q_{2}(2|p)=Q_{2}(p-1|3)\;(B-21)$$

### 3.4. 4 Particles

For *n* = 4, there are the following cases:

**(Case 1)** $k_{1}$ lies in the uppermost, first from above, energy interval:

(25) $$\left(p-1\right)\frac{4\kappa }{p}\leq k_{1}\leq p\frac{4\kappa }{p}=4\kappa$$

By virtue of the constraint $k_{2}+k_{3}\leq 4\kappa -k_{1}$ (16), $k_{2}$ and $k_{3}$ lie in the lowest energy interval, and $k_{4}$ is always fixed by virtue of the constraint (16).

(26) $$0\frac{4\kappa }{p}=0\leq k_{2},k_{3}\leq 1\frac{4\kappa }{p}=\frac{4\kappa }{p}$$

This represents one possibility for *k*_2_$k_{2}$ and *k*_3_.

**(Case 2)** $k_{1}$ lies in the second-highest energy interval, as follows:

(27) $$\left(p-2\right)\frac{4\kappa }{p}\leq k_{1}\leq \left(p-1\right)\frac{4\kappa }{p}\;(B-22)$$

By virtue of the constraint $k_{2}+k_{3}\leq 4\kappa -k_{1}$ (16), again, $\left(k_{2},k_{3}\right)$ may lie in the lowest or in the second-lowest intervals, (1,1), (1,2), or (2,1), while (2,2) is energetically not accessible. The sum of the interval numbers is not larger than three. This represents three possibilities for $k_{2}$ and $k_{3}$.

**(Case 3)** $k_{1}$ lies in the third-highest energy interval: By the constraint $k_{2}+k_{3}\leq 4\kappa -k_{1}$ (16), again, $\left(k_{2},k_{3}\right)$ may lie in the lowest, second-lowest or third-lowest intervals, (1,1), (1,2), (1,3), (2,1), (2,2), or (3,1). The sum of the interval numbers is not larger than four. This represents six possibilities for $k_{2}$ and $k_{3}$.

**(General case)** In the general case, we have the following:

(28) $$\left(p-q\right)\frac{4\kappa }{p}\leq k_{1}\leq \left(p-q+1\right)\frac{4\kappa }{p}\;(B-24)$$

There are $q\left(q+1\right)/2$ different possibilities for $k_{2}$ and $k_{3}$. This is easily seen when one considers not the possibilities for $k_{2}$ and $k_{3}$ but that for $k_{2}$ and $\left(k_{2}+k_{3}\right)$. For them, one obtains (1,1) for case 1 above, (1,1), (1,2), (22) for case 2, and (1,1), (1,2), (1,3), (2,2), (2,3), (3,3) for case 3. There are no other combinations of two elements out of the 1, 2, and 3 elements, respectively. For case 3, this means that one has an urn with three numbered balls, selects one ball, notes its number, and puts it back into the urn. Then, one selects a ball for a second time and notes its number. The six possible combinations above are the possible outcomes, where the sequence of the numbers is discarded, i.e., the outcome (2,1) is considered to be the same as the outcome (1,2). This is the urn model with *q* balls, two drawings with repetition, and without accounting for the sequence. The general number of different outcomes for *q* balls and *r* drawings equals $\left(\begin{array}{c} q+r-1\\ q-1 \end{array}\right)$. Here, r=2 and $\left(\begin{array}{c} q+r-1\\ q-1 \end{array}\right)=\left(\begin{array}{c} q+1\\ q-1 \end{array}\right)=\frac{q\left(q+1\right)}{2}$ as obtained by Boltzmann on p. 85.

For all possible *p* intervals for $k_{1}$, the number of different possibilities for $k_{2}$ and $k_{3}$ sum up to the following number of different distributions (p. 85):

(29) $$\frac{1\cdot 2}{2}+\frac{2\cdot 3}{2}+\ldots +\frac{p\left(p+1\right)}{2}=\frac{p\left(p+1\right)\left(p+2\right)}{2\cdot 3}=Q_{2}(3|p)=Q_{2}(p-1|4)\;(B-25)$$

### 3.5. n Particles. Summary and Discussion

The Formulae (18), (20), (24) and (29) for the number of different distributions for n=1,2,3,4 particles generalize to the following. There are the following number of different distributions of the *p* energy parts (particles), *ε*, onto the *n* molecules (cells):

(30) $$\frac{p\left(p+1\right)\cdots \left(p+n-2\right)}{2\cdot 3\cdots (n-1)}=\frac{\left(p+n-2\right)!}{\left(p-1\right)!(n-1)!}=\left(\begin{array}{c} p+n-2\\ n-1 \end{array}\right)=Q_{2}(n-1|p)=Q_{2}(p-1|n)$$

This differs from $Q_{2}(n|d)\hat{=}Q_{2}(p|n)$ (7). Boltzmann stresses (see above) that there are only n−1 independent occupation numbers because $k_{n}=p\varepsilon -k_{1}-k_{2}-\cdots -k_{n-1}$ is determined by the first n−1 numbers, $k_{1},k_{2},\cdots ,k_{n-1}$. However, the amount of the six different occupation number vectors in Scheme 2 above is correctly calculated using $Q_{2}(p|n)$ (7).

Bach writes that there is not *p*, but only p−1 energy parts, because Boltzmann “does not include particles of zero energy” [3] (p. 11, fn. 64). This contradicts Boltzmann’s introduction of the *p* energy parts as well as the presence of *p* energy intervals in the list (19). Possibly, Bach’s argument stems from Boltzmann’s 1877 discretization (38) of the energy spectrum.

Boltzmann’s and Bach’s arguments refer to the two mathematically equivalent forms of $Q_{2}$ in formula (30). This shows, again, that the constraint (17) makes them physically *not* equivalent.

For large values of *n* and *p*, the difference in the $Q_{2}$ used by Planck (1900c-2)/(106) is negligible. This suggests the entropy of this model system to equal

(31) $$S_{p}(n)=k_{B}\ln\left\{Q_{2}(n-1|p)\right\}+const$$

For large values of *n* and *p*, this expression is equivalent to Boltzmann’s 1877 possible entropy (85) and Planck’s 1901 entropy (1901-6)/(121).

Notice that Boltzmann in 1868 did not yet have that understanding of the interrelation between entropy and probability. Nevertheless, he was already far ahead progressed in this memoir at age 24.

If, furthermore, the fundamental formula (89) had been known to Boltzmann, he could have proceeded as follows:

(32) $$\frac{1}{k_{B}T}=\frac{d}{dE}\ln\left\{Q_{2}(n-1|p)\right\}\approx \frac{1}{\varepsilon }\ln\left(\frac{n}{p}+1\right);n\gg 1,p\gg 1$$

Hence, the average number of energy portions and, thus, energy per particle equals the following:

(33) $$\frac{p}{n}=\frac{1}{e^{\frac{\varepsilon }{k_{B}T}}-1};U=\frac{p\varepsilon }{n}=\frac{\varepsilon }{e^{\frac{\varepsilon }{k_{B}T}}-1}$$

This is Planck’s result for the average energy of a resonator, *U*; of course, Boltzmann had no reason to specify ε=hν. As long as *ε* is finite, Boltzmann deals not with classical, but quantum statistics.

Admirably enough, Boltzmann calculates the probability, $P_{n}\left(k\right)$, that the kinetic energy, *k*, of one of the *n* particles lies between *k* and k+dk, as follows (p. 85)^6^:

(34) $$P_{n}(k)=\frac{\left(n-1\right){\left(n\kappa -k\right)}^{n-2}}{{\left(n\kappa \right)}^{n-1}}dk=\frac{\left(n-1\right){\left(1-\frac{k}{n\kappa }\right)}^{n-2}}{n\kappa }dk\begin{array}{ccc} & \underset{n\to \infty }{\to } & \end{array}\frac{1}{\kappa }e^{-\frac{k}{n\kappa }}dk\;(B-26,27)$$

This is the same expression he obtains from the following (p. 86):

(35) $$P_{n}(k)=\frac{dk_{1}\int_{0}^{n\kappa -k}dk_{2}\int_{0}^{n\kappa -k_{1}-k_{2}}dk_{3}\cdots \int_{0}^{n\kappa -k_{1}-k_{2}-\cdots -k_{n-2}}dk_{n-1}}{\int_{0}^{n\kappa }dk_{1}\int_{0}^{n\kappa -k_{1}}dk_{2}\int_{0}^{n\kappa -k_{1}-k_{2}}dk_{3}\cdots \int_{0}^{n\kappa -k_{1}-k_{2}-\cdots -k_{n-2}}dk_{n-1}}\;(B-27)$$

“Compared to later developments, which are (following Planck) based on unnormalized “probabilities”, these two pages … [pp. 84, 85] are a masterpiece in probability theory. Boltzmann calculates the combinatorial formula, equ. (32) [(30)], for which EHRENFEST & KAMERLINGH-ONNES give their well-known derivation some fifty years later.^7^ He evaluates the marginal distribution (PÓLYA distribution [19]) by replicating his previous argument and, in the continuum limit, obtains a scaled β-distribution. Finally, in the macroscopic limit, he derives the exponential distribution.” [3] (p. 14).

## 4. Boltzmann’s 1877 State Definition and Counting

“Barely any of Boltzmann’s original scientific work is available in translation.^8^ This is remarkable given his central role in the development of both equilibrium and non-equilibrium statistical mechanics, his statistical mechanical explanation of entropy, and our understanding of the Second Law of thermodynamics. What Boltzmann actually wrote on these subjects is rarely quoted directly, his methods are not fully appreciated, and key concepts have been misinterpreted.^9^ Yet his work remains relevant today.” [7] (p. 1971f).

The 1877 memoir exemplifies several of Boltzmann’s most important contributions to modern physics. These include the eponymous Boltzmann distribution, much of the theoretical apparatus of statistical mechanics, and the statistical mechanical formulation of entropy.

Boltzmann’s “permutability measure”, Ω (3/2 of Clausius’ entropy, *S*), is constructed as an *extensive* quantity. Thus, Boltzmann never encounters the apparent Gibbs paradox for the entropy of mixing identical gases. 

Last, but not least, Boltzmann’s statistical definition of entropy is the first one that applies to non-equilibrium states, thus “opening the door to the statistical mechanics of non-equilibrium states and irreversible processes.” [7] (p. 1974).

### 4.1. The Discrete Gas Model

In his 1872 memoir [23], Section II, Boltzmann introduces occupancy numbers. However, he does not consider their probability distribution and their most probable values as he does in 1877. For a valuation of the 1872 memoir, the reader is referred to [3] (chp. 4).

For simplicity, Boltzmann begins (p. 1976)^10^ with an ideal gas model, in which each of the *n* molecules can assume only “a *finite* number of velocities, *v*, such as

(36) $$v=0,\frac{1}{q},\frac{2}{q},\frac{3}{q},\ldots \frac{p}{q}\;(B-0+1)$$

where *p* and *q* are arbitrary finite numbers.” (p. 1976; (B-0+*n*) enumerates Boltzmann’s *n*^th^ formula before his first formula, (B-1)). Accordingly, the kinetic energy, $E_{\mathrm{kin}}$, of each molecule of mass, *m*, can also assume only a *finite* number of values.

(37) $$E_{\mathrm{kin}}=0,\frac{m}{2}{\left(\frac{1}{q}\right)}^{2},\frac{m}{2}{\left(\frac{2}{q}\right)}^{2},\frac{m}{2}{\left(\frac{3}{q}\right)}^{2},\ldots \frac{m}{2}{\left(\frac{p}{q}\right)}^{2}$$

Now, when considering solely the distribution of the various values of $E_{\mathrm{kin}}$ over the molecules, it is simpler to assume for them an arithmetic progression of p+1 (kinetic) energy levels.

(38) $$\left\{E_{\mathrm{kin}}\right\}=\left\{0,\varepsilon ,2\varepsilon ,3\varepsilon ,\ldots p\varepsilon \right\}$$

In what follows, I will present lengthy quotations to find out which level of Section 2 Boltzmann’s complexions finally belong to (he switches between the three levels). Luckily, Boltzmann discards the mechanical details of the energy exchange (collisions) and concentrates on the combinatorial–probabilistic side of the model.

### 4.2. The Kinetic Energy Distribution. Complexions

“If we know how many of these *n* molecules have a kinetic energy of zero [say, $z_{0}$], how many have a kinetic energy of *ε* $\left[z_{1}\right]$ and so on, then we know the kinetic energy distribution.” (p. 1977).

This wording of the occupancy numbers, $z_{0}$, $z_{1}$, *etc*. corresponds to Level 3, as shown in Section 2.3 and our discussion of constraints (40) and (41).

The energies (38) are distributed in all possible ways among the *n* molecules so that the total energy is constant.

(39) $$E_{\mathrm{kin},\mathrm{tot}}=L=\lambda \varepsilon =const,$$

where Boltzmann’s “*L*” refers to “lebendige Kraft” (living force, see the remark before Equation (16). This represents a *constraint*, which will show up in Equation (41). The combinatorial scheme is this:^11^

$$n\;\mathrm{particles}\;\mathrm{in}\;d\;\mathrm{cells}\;\hat{=}\lambda \mathrm{energy}\mathrm{portions}\mathrm{on}n\mathrm{molecules}$$

“Any such distribution, in which the first molecule may have a kinetic energy of e.g., $\left[k_{1}\varepsilon =\right]2\varepsilon$, the second may have $\left[k_{2}\varepsilon =\right]6\varepsilon$, and so on, up to the last molecule, we call a *complexion*, and so that each individual complexion can be easily enumerated, we write them in sequence (for convenience we divide through by *ε*), specifying the kinetic energy of each molecule. We seek the number P of complexions where *w*_0_ molecules have kinetic energy 0, $w_{1}$ molecules have kinetic energy *ε*, $w_{2}$ have kinetic energy 2*ε*, up to the $w_{p}$ which have kinetic energy *pε*. We said, earlier, that given how many molecules have kinetic energy 0, how many have kinetic energy *ε*, etc., this distribution among the molecules specifies the number of P of complexions for that distribution; in other words, it determines the likelihood of that state distribution. Dividing the number P by the number of all possible complexions, we get the probability of the state distribution.” (p. 1977).

Therefore, $w_{0},w_{1},w_{2},\cdots w_{p}$ are the occupancy numbers of Level 3 (see Section 2.3), while $k_{1}$, $k_{2}$, *etc*. are the occupation numbers of Level 2 (see Section 2.2). A complexion is thus a occupation number vector, **k**, while a “state distribution” is an occupancy number vector, **w**. However, the occupation numbers (complexions) are *not* uniquely determined by an occupancy number vector (state distribution).

“It is now immediately clear that the number P for each state distribution is exactly the same as the number of permutations of which the elements of the state distribution are capable, and that is why the number P is the desired measure of the permutability of the corresponding distribution of states. Once we have specified every possible complexion, we have also all possible state distributions, the latter differing from the former only by immaterial permutations of molecular labels.” (p. 1977).

Indeed, for each given complexion (occupation number vector, **k**), the corresponding state distribution (occupancy number vector, **w**), can be calculated using formula (13). The “immaterial permutations” refer to the interchangeability of the molecules (cells) carrying the same number of energy portions, *ε*.

However, “immaterial” may be misleading. For this, I propose this following translation: “For if we once think of all possible complexions written down, and then also of all possible distributions of states, the latter will differ from the former only by the fact that in them it is indifferent at which place the numbers stand.” This refers to the (non-)interchangeability of the cells as mentioned in the foregoing paragraph. In other words, as Boltzmann continues,

“All those complexions which contain the same number of zeros, the same number of ones etc., differing from each other merely by different arrangements of elements, will result in the same state distribution; the number of complexions forming the same state distribution, and which we have denoted by P, must be equal to the number of permutations which the elements of the state distribution are capable of.” (p. 1977f).

In modern terms, Boltzmann considers the occupation number vectors (complexions) as microstates and the occupancy number vectors as macrostates. This differs from Planck’s treatment (see Section 6), but may also lead to a Planckian distribution formula, as shown in Section 4.8.

Schöpf [24] (p. 70) writes that a complexion describes the particles are in each cell, i.e., a configuration. On p. 116, he correctly writes that Boltzmann’s macrostate corresponds to the number of resonators with energy, *rε* (occupancy). On p. 125, he returns to the erroneous conclusion that Boltzmann’s “definition of the microstate comes from the question, *which* particles are in the *n*^th^ state [cell, i.e., configurations], and therefore fundamentally presupposes their distinguishability.” In a configuration, the particles are distinguishable, i.e., not interchangeable, indeed, see Section 2.1. Boltzmann, however, works with occupations, in which the particles are indistinguishable, i.e., interchangeable, see Section 2.2. Moreover, we will see in Section 4.8 that Boltzmann’s approach does lead to a Planckian distribution law if it is properly finished. Furthermore, the probability (B-3)/(42) (being equivalent to $Q_{3\;}$(15)) depends solely on the number of particles and the occupancy numbers. The result of its maximization is, thus, independent of the representation of the microstates through configurations or occupations.

In contrast to his 1868 memoir [5], Boltzmann tackles a *non*-equilibrium theory. This requires the comparison of the most probable distribution with neighboring ones [17] (p. 20). This comparison is possible when working with occupancy numbers, because the “thermodynamic probabilities” (15) (Level 3) and (B-3)/(42) below, which determine the entropy, depend on them. In contrast, the “thermodynamic probabilities” (7) (Level 2) and (30) (Boltzmann 1868 [5]) are *in*dependent of the occupations (distributions), **k**. Consequently, the entropy can*not* be maximized with respect to neighboring distributions.

### 4.3. Example

As an example, Boltzmann considers the case n=λ=p=7. I will sketch it here to avoid confusion between the occupancy numbers, $\left(w_{0},\cdots ,w_{7}\right)$, in Boltzmann’s text and the occupation numbers, $\left(k_{1},\cdots ,k_{7}\right)$, in Boltzmann’s table (Table 1 below).

“With 7 molecules, there are 8 possible values for the kinetic energy, 0, *ε*, 2*ε*, 3*ε*, 4*ε*, 5*ε*, 6*ε*, 7*ε* to distribute in any possible way such that the total kinetic energy = 7*ε*. There are then 15 possible state distributions. We enumerate each of them in the above manner, producing the numbers listed in the second column of the following table of state distributions (Table 1). The numbers in the first column label the different state distributions.” (p. 1978).

“…P is the number of possible permutations of members for each state. The first state distribution, for example, has 6 molecules with zero kinetic energy, and the seventh has kinetic energy 7*ε*. So $w_{0}=6$, $w_{7}=1$, $w_{1}=w_{2}=w_{3}=w_{4}=w_{5}=w_{6}=0$. [See Table 2; The author had added $w_{1}$.] It is immaterial which molecule has kinetic energy 7ε. So there are 7 possible complexions which represent this state distribution. Denoting the sum of all possible complexions, 1716, by *J* then the probability of the first state distribution is 7/J; similarly, the probability of the second state distribution is 42/J; the most probable state distribution is the tenth, as its elements permit the greatest number of permutations. Hereon, we call the number of permutations the relative likelihood of the state distribution…” (p. 1978). 

For the reader’s convenience, The author had added Table 2 which contains the occupancy numbers, $\left(w_{0},\cdots ,w_{7}\right)$, in Boltzmann’s text. 

The urn model then presented by Boltzmann is an illustration, which, for this contribution, does not provide new insights, cf. his 1868 counting [5] considered in Section 3, and also [17] (p. 16, fn. 53), [25] (pp. 290–291).

### 4.4. The Number of Complexions, P

“We would first like to calculate the permutations P for the state distribution characterized by $w_{0}$ molecules with kinetic energy 0, $w_{1}$ molecules with kinetic energy *ε*, etc. It must be understood that

(40) $$w_{0}+w_{1}+w_{2}+\ldots +w_{p}=n\;(B-1)$$

(41) $$w_{0}\cdot 0+w_{1}\cdot 1+w_{2}\cdot 2+\ldots +w_{p}\cdot p=\lambda \;(B-2)$$

because the total number of molecules is *n*, and the total kinetic energy is λε=L.” (p. 1979).

The existence of these two constraints confirms the vector $w=\left(w_{0},\cdots ,w_{p}\right)$ to be an occupancy number vector. To see that, set p=λ and compare them with constraint (14) in Section 2.3. Recall that the number of molecules, *n*, corresponds to the number of cells, and that the number of energy portions, *λ*, corresponds to the number of particles. The exact number of possible values of kinetic energy, p+1, is actually rather irrelevant, since the combinatorics is about the distribution of the *λ* energy portions onto the *n* molecules. If p<λ, one has $w_{p+1}=\cdots =w_{\lambda }\equiv 0$. For this, and for the sake of full compatibility with the three description levels in Section 2 (configuration–occupation–occupancy), which are used by Boltzmann and Planck, it is best to set p=λ, or p=∞. Boltzmann does the latter in his Section IV, p. 2001ff.

This conclusion regarding **w** agrees with Boltzmann’s semantic formulation of the definition of **w**, viz., ‘how many molecules *aka* cells host 0, 1, … *p* energy elements *aka* particles?’, as discussed at the beginning of Section 2.

“Describing the state distribution as before, a complexion has $w_{0}$ molecules with zero energy, $w_{1}$ with one unit, and so on.^12^ The permutations, P, arise since of the *n* elements $w_{0}$ are mutually identical. Similarly with the $w_{1}$, $w_{2}$, etc. elements. The total number of [that] permutations is well known…” (p. 1979)

(42) $$P=\frac{n!}{w_{0}!w_{1}!\ldots }\;\cdot (B-3)$$

### 4.5. The Most Likely State Distribution

“The most likely state distribution will be for those $w_{0}$, $w_{1}$, … values for which P is a maximum or since the numerator is a constant, for which the denominator is a minimum. The values $w_{0}$, $w_{1}$ [etc.] must simultaneously satisfy the two constraints (1) [(40)] and (2) [(41)]. Since the denominator of P is a product, it is easiest to determine the minimum of its logarithm, that is the minimum of” (p. 1979) the following:

(43) $$M=ln(w_{0}!)+ln(w_{1}!)+\ldots \;(B-4)$$

Applying Stirling’s formula to P (B-3)/(42) and accounting for the huge number of molecules, yields

(44) $$M=\sum_{s=0}^{p}w_{s}\ln w_{s}$$

“The work of Boltzmann has clarified the multifaceted significance of this function.” [26] (p. 39, 12d)—In view of the fact that Boltzmann 1868 [5] has used occupation numbers, but here—occupancy numbers, is it misleading to write that *M* “derives from the same combinatorial analysis developed by Boltzmann in 1868.” [17] (p. 20).

Boltzmann applies the method of Lagrangian multipliers to account for constraints (40) and (41). (I abbreviate Boltzmann’s calculations). Therefore, the most probable occupancy number vector is

(45) $$w=\left(w_{0},w_{1},\cdots ,w_{p}\right)=\min\left\{\tilde{M}\right\};\tilde{M}:=\sum_{r=0}^{p}w_{r}\ln w_{r}+h\left(\sum_{r=0}^{p}w_{r}-n\right)+k\left(\sum_{r=0}^{p}r\cdot w_{r}-\lambda \right)$$

The minimum is found by equating all of the derivatives of $\tilde{M}$ equal to zero, where the derivatives with respect to *h* and *k* merely reproduce constraints (40) and (41), respectively.

(46) $$\begin{array}{c} \frac{\partial \tilde{M}}{\partial w_{0}}=ln(w_{0})+1+h+0\cdot k=0,\frac{\partial \tilde{M}}{\partial w_{1}}=ln(w_{1})+1+h+1\cdot k=0,\\ \frac{\partial \tilde{M}}{\partial w_{2}}=ln(w_{2})+1+h+2\cdot k=0,\begin{array}{ccc} & \cdots & \end{array}\frac{\partial \tilde{M}}{\partial w_{p}}=ln(w_{p})+1+h+p\cdot k=0 \end{array}$$

A simple calculation yields

(47) $$w_{s}=e^{-1-h-ks}=w_{0}e^{-ks}=w_{0}{\left(\frac{w_{1}}{w_{0}}\right)}^{s},s=0,1,\cdots ,p,$$

or

(48) $$1+h=\ln\frac{1}{w_{0}},k=-\ln\frac{w_{1}}{w_{0}};\frac{w_{s}}{w_{0}}={\left(\frac{w_{1}}{w_{0}}\right)}^{s},s=0,1,\cdots ,p$$

Inserting the rightmost formula into constraints (40) and (41) leads to

(49) $$\frac{n}{w_{0}}=\sum_{s=0}^{p}x^{s}=\frac{1-x^{p+1}}{1-x};\Rightarrow x^{p+1}-\frac{n}{w_{0}}\cdot x+\frac{n}{w_{0}}-1=0;x:=\frac{w_{1}}{w_{0}}\;(B-10)$$

and

(50) $$\begin{array}{ll} \begin{array}{l} \frac{\lambda }{w_{0}}=\sum_{s=0}^{p}sx^{s}=\frac{x+\left(px-p-1\right)x^{p+1}}{{\left(1-x\right)}^{2}};x:=\frac{w_{1}}{w_{0}}\Rightarrow \\ px^{p+2}-\left(p+1\right)x^{p+1}-\frac{\lambda }{w_{0}}x^{2}+\left(1-2\frac{\lambda }{w_{0}}\right)x-\frac{\lambda }{w_{0}}=0 \end{array} & \left(B-11\right) \end{array}$$

Combining both equations yields the following:

(51) $${\tilde{w}}_{1}^{2}+\frac{p\left(1-\tilde{n}\right)-\left(1+p\right)\tilde{n}+1-2\tilde{\lambda }}{p\tilde{n}-\tilde{\lambda }}{\tilde{w}}_{1}-\frac{\tilde{\lambda }+\left(1+p\right)\left(1-\tilde{n}\right)}{p\tilde{n}-\tilde{\lambda }}=0;\begin{array}{cc} {\tilde{w}}_{1}:=\frac{w_{1}}{w_{0}}, & etc. \end{array}$$

This provides an explicit formula for $w_{1}/w_{0}$ in terms of *p*, $n/w_{0}$, and $\lambda /w_{0}$. Boltzmann, however, goes another way.

By dividing (B-11)/(50) by (B-10)/(49), Boltzmann obtains

(52) $$\left(pn-\lambda \right)x^{p+2}-\left(pn+n-\lambda \right)x^{p+1}+\left(n+\lambda \right)x-\lambda =0\;(B-12)$$

“One can see immediately from Descartes’ theorem^13^ that this equation cannot have more than three real positive roots, of which two are = 1. Again it is easy to see that both roots are not solutions of Equations (8) and (9), and also do not solve the problem, but that they showed up in the final equation merely as a result of multiplying by the factor ${\left(x-1\right)}^{2}$ [see the denominator in formula (50)].” (p. 1982).

In bypassing, I notice that one can introduce an analogue to the canonical partition function, *Z* (cf. [24], formula (15)).

(53) $$w_{s}=\frac{n}{Z}e^{-ks};Z:=\sum_{s=0}^{p}e^{-ks},$$

where $e^{-ks}$ is an analogue of the Boltzmann factor. 

“We note again that the largest allowed kinetic energy, P=pε, is very large compared to the mean kinetic energy of a molecule” (p. 1982).

(54) $$P=p\varepsilon \gg \frac{L}{n}=\frac{\lambda \varepsilon }{n}\begin{array}{ccc} & \Rightarrow & \end{array}p\gg \frac{\lambda }{n},pn\gg \lambda$$

As $0<x\equiv w_{1}/w_{0}<1$ and p≫1, the terms with the (*p*+2)^th^ and (*p*+1)^th^ power in Equation (52) can be neglected. This yields

(55) $$x=\frac{\lambda }{n+\lambda }=\frac{w_{1}}{w_{0}};p\gg 1\;(B-13+3)$$

((B-*k*+*n*) enumerates the *n*th formula after Boltzmann’s *k*th formula, (B-*k*)). The left formula (B-10)/(49) is equivalent to

(56) $$w_{0}=\frac{1-x}{1-x^{p+1}}n\;(B-14)$$

Finally, for very large values of *p*, the occupancy numbers assume the “limiting values”

(57) $$w_{0}=\frac{n^{2}}{n+\lambda },w_{1}=\frac{n^{2}\lambda }{{\left(n+\lambda \right)}^{2}},w_{2}=\frac{n^{2}\lambda^{2}}{{\left(n+\lambda \right)}^{3}}etc.\;(B-15)$$

“It is seen from the quotients…”,

(58) $$\frac{w_{s}}{n}=\frac{n}{n+\lambda }\cdot {\left(\frac{\lambda }{n+\lambda }\right)}^{s}=\frac{1}{1+\frac{\lambda }{n}}\cdot {\left(\frac{\frac{\lambda }{n}}{1+\frac{\lambda }{n}}\right)}^{s};s=0,1,\ldots ,p,$$

“…that the probabilities of the various kinetic energy values [$w_{s}/n$] for larger *p* are again dependent almost exclusively on the mean energy of the molecule [λ/n]” (p. 1982f). This is a truncated geometric distribution with a mean of λ/n for the normalized occupancy numbers, $w_{s}/n$ [3] (p. 20).

Notice that the approximation (55) is compatible with constraints (40) and (41) as follows:

(59) $$\begin{array}{c} \sum_{s=0}^{p}w_{s}=\frac{n^{2}}{n+\lambda }\sum_{s=0}^{p}{\left(\frac{\lambda }{n+\lambda }\right)}^{s}=n-n{\left(\frac{\lambda }{n+\lambda }\right)}^{p}\begin{array}{ccc} & \underset{p\to \infty }{\to } & \end{array}n;\\ \sum_{s=0}^{p}sw_{s}=\frac{n^{2}}{n+\lambda }\sum_{s=0}^{p}s{\left(\frac{\lambda }{n+\lambda }\right)}^{s}=\lambda +\left(\frac{n\lambda }{n+\lambda }+n-1\right){\left(\frac{\lambda }{n+\lambda }\right)}^{p+1}\begin{array}{ccc} & \underset{p\to \infty }{\to } & \end{array}\lambda \end{array}$$

Boltzmann shows that this approximation works well, even for such small numbers as n=p=λ=7 (cf. also Table 2 above). Moreover, he shows that the values (B-15)/(57) of $w_{s}$ belong to a minimum of *M* (44).

The absolute probability equals *W* = P/*J*, where *J* is “the sum of the permutations P for all possible state distributions” (p. 1979). “One easily finds that *J* is given by the following binomial coefficient”:

(60) $$J=\left(\begin{array}{c} \lambda +n-1\\ \lambda \end{array}\right)\equiv \frac{\left(\lambda +n-1\right)!}{\lambda !\left(n-1\right)!}\;(B-15+3)$$

(p. 1983). It equals the number of occupation number vectors, $Q_{2}(\lambda |n)$ (7), for *λ* particles (here referred to as energy portions, *ε*) in *n* cells (molecules). This confirms the statement above, that Boltzmann considered the occupation as a microstate and the occupancy as a macrostate.

With the relative probability, P (B-3)/(42), the absolute probability equals

(61) $$\frac{P}{J}=\frac{n!}{w_{0}!w_{1}!\ldots }{\left(\begin{array}{c} \lambda +n-1\\ \lambda \end{array}\right)}^{-1}=P_{\mathrm{BE}}(w)$$

(see [4], p. 133, formula (5.6)). This means that, in this part of his 1877 memoir [2], Boltzmann is implicitly performing Bose–Einstein, or, more accurately, Planck statistics (there is no chemical potential), as shown in Formula (68).

“According to Equation (15) [(57)], the probability of having a kinetic energy *sε* is given by [see Formula (58)]

(62) $$w_{s}=\frac{n^{2}}{n+\lambda }\cdot {\left(\frac{\lambda }{n+\lambda }\right)}^{s}\;(B-18+5)$$

since.^14^ This is logically more stringent) λε/n is equal to the average kinetic of a molecule *µ*, which is finite, so *n* is very small compared to *λ*. So the following approximations

(63) $$\frac{n^{2}}{n+\lambda }=\frac{n^{2}}{\lambda }=\frac{n\varepsilon }{\mu },\frac{\lambda }{n+\lambda }=1-\frac{n}{\lambda }=e^{-\frac{n}{\lambda }}=e^{-\frac{\varepsilon }{\mu }}\;(B-18+6)$$

hold. From which is follows that

(64) $$w_{s}=\frac{n\varepsilon }{\mu }e^{-\frac{s\varepsilon }{\mu }}."\;\left(p.\;1986\right)\;(B-18+8)$$

Notice that the calculation of $w_{s}$ can largely be simplified [27] (p. 251). Accounting for the constraints (B-1)/(40) and (B-2)/(41), the minimum of *M* (44) is determined using the following three equations:

(65) $$\delta M=\delta \sum_{s=0}^{p}w_{s}\ln w_{s}=\sum_{s=0}^{p}\left(\ln w_{s}+1\right)\delta w_{s}=0;\sum_{s=0}^{p}\delta w_{s}=0;\sum_{s=0}^{p}\varepsilon_{s}\delta w_{s}=0$$

where arbitrary energy levels, $\varepsilon_{s}$, and infinitesimal variations, $\delta w_{s}$, are allowed. The solution of them is

(66) $$w_{s}=c_{1}e^{-c_{2}\varepsilon_{s}};c_{1,2}=const.$$

The constants are to be calculated by employing the constraints (B-1)/(40) and (B-2)/(41). For equidistant energy levels, $\varepsilon_{s}=s\varepsilon$ (38), and a very large *p*, one recovers Boltzmann’s formula (62):

(67) $$c_{1}=\frac{n^{2}}{n+\lambda },e^{-c_{2}\varepsilon }=\frac{\lambda }{n+\lambda },w_{s}=c_{1}e^{-c_{2}s\varepsilon }=n\frac{1}{1+\frac{\lambda }{n}}{\left(\frac{\frac{\lambda }{n}}{1+\frac{\lambda }{n}}\right)}^{s}$$

Then, the average energy of a molecule becomes [27] (p. 254)

(68) $$U=\varepsilon \frac{\sum_{s=0}^{p}sw_{s}}{\sum_{s=0}^{p}w_{s}}=\varepsilon \frac{1}{e^{c_{2}\varepsilon }-1}\left(\frac{1+\left(pe^{-c_{2}\varepsilon }-p-1\right)e^{-c_{2}\varepsilon p}}{1-e^{-c_{2}\varepsilon \left(p+1\right)}}\right)\begin{array}{ccc} & \underset{p\to \infty }{\to } & \end{array}\frac{\varepsilon }{e^{c_{2}\varepsilon }-1}$$

This is a Planckian result, as shown in Section 4.8.

In the following Section II, Boltzmann considers a transition to the continuum. Section III deals with polyatomic gas molecules and external forces using both the discrete theory of Section I and the continuum theory of Section II.

### 4.6. Unfinished Combinatorics about the Most Likely State Distribution

In Section IV, Boltzmann returns to combinatorics to demonstrate “how general^15^ the concept of the most probable state distribution of gas molecules is.” (p. 2001). He chooses a different urn model to that on p. 1978. 

“We have in an urn just as many identical balls (*n*) as molecules present. Every ball corresponds to a certain molecule.^16^ We now make *λ* draws from this urn, returning the ball to the urn each time. The kinetic energy of the first molecule is now equal to the product of *ε* and the number of times the ball corresponding to this molecule is drawn. The kinetic energies of all other molecules are determined analogously. We have produced a distribution of the kinetic energy *L* among the molecules (a *complexion*).” (p. 2001). The number of drawings corresponds to the occupation numbers; a complexion is an occupation number vector, again. 

This is carried out *J* times, yielding *J* complexions. The most likely state distribution can be found in two ways.

#### 4.6.1. Bernoulli (Binomial) Distribution

“First, we find how often in all *J* complexions a molecule has kinetic energy 0, how often the kinetic energy is *ε*, 2*ε*, etc., and say that the ratios of these numbers should provide the probabilities that a molecule has kinetic energy 0, *ε*, 2*ε*, etc. at thermal equilibrium. … the probability that the first molecule was picked in the first draw is 1/n; however, the probability that another ball was drawn is $\left(n-1\right)/n$. Thus, the probability that on the 1st, 2nd, 3rd … *k*th draws the molecule corresponding to the first ball has been picked, and then a different ball for each of the following is given by

(69) $${\left(\frac{1}{n}\right)}^{k}\cdot {\left(\frac{n-1}{n}\right)}^{\lambda -k}={\left(\frac{n-1}{n}\right)}^{\lambda }\cdot {\left(\frac{1}{n-1}\right)}^{k}\;(B-60b+1)$$

Likewise is the probability that the ball corresponding to the first molecule is picked on the 1st, 2nd, 3rd … (*k*−1)th, and then (*k*+1)th draws etc. The probability that the ball corresponding to the first molecule is picked for any arbitrary *k* draws, and not for the others is…” (p. 2002)

(70) $$w_{k}=\frac{\lambda !}{\left(\lambda -k\right)!k!}\cdot {\left(\frac{n-1}{n}\right)}^{\lambda }\cdot {\left(\frac{1}{n-1}\right)}^{k}\;(B-60b+2)$$

Notice that this $w_{k}$ is not an occupancy number. 

Boltzmann, approximating the faculty functions using a form of Stirling’s formula, obtains an expression, “which shows that the probability of the larger kinetic energies is so disproportionately important that the entire expression does not approach a clearly identifiable limit with increasing *k*, *λ*, 1/*ε* and *n*.” (p. 2002). 

Actually, $w_{k}$ (70) equals the probability $b\left(k;\lambda ,\frac{1}{n}\right)$, that *k* Bernoulli trials with a probability of $\frac{1}{n}$ for success and of $1-\frac{1}{n}$ for failure result in *k* successes and n−k failures. [13] (p. 148, theorem with formula (2.1)). Thus, Boltzmann has reproduced the Bernoulli distribution. For large values of *λ*, the classical Poisson approximation, or Poisson limit theorem, reads as follows [28], [13] (p. 154, formula (5.6)):

(71) $$w_{k}=b(k;\lambda ,\frac{1}{n})\approx \frac{1}{k!}{\left(\frac{\lambda }{n}\right)}^{k}e^{-\frac{\lambda }{n}};n\gg 1,\frac{\lambda }{n}∼1$$

The approximation is the better, the larger *λ*, when *λ*/*n* remains of moderate magnitude. 

This means that Boltzmann’s claim that there is no “clearly identifiable limit with increasing *k*, *λ*, 1/*ε* and *n*” (p. 2002) is not correct. Nevertheless, this “method of probability determination” does not lead to the correct result.

#### 4.6.2. The Most Probable State Distribution. II. Maxwell-Boltzmann à la Bach

Second, Boltzmann considers “all *J* complexions that we have formed by *J* drawings of *λ* balls from our urn. One of the various possible complexions consists of *λ* drawings of the ball corresponding to the first ball.^17^ We want to express this complexion symbolically by^18^

(72) $$m_{1}^{\lambda }\cdot m_{2}^{0}\cdot m_{3}^{0}\ldots \cdot m_{n}^{0}$$

A second complexion, with *λ* − 1 draws of the ball corresponding to the first molecule, and one draw of the ball corresponding to the second molecule we want to express as

(73) $$m_{1}^{\lambda -1}\cdot m_{2}^{1}\cdot m_{3}^{0}\ldots \cdot m_{n}^{0}\;(B-60b+4)$$

We see that the different possible complexions are expressed exactly by various components; the sum of these appears as the power series

(74) $${\left(m_{1}+m_{2}+m_{3}+\ldots +m_{n}\right)}^{\lambda }\;(B-A)$$

that is developed according to the polynomial theorem.” (p. 2002) The latter one reads as follows:

(75) $${\left(m_{1}+m_{2}+\ldots +m_{n}\right)}^{\lambda }=\sum_{\begin{array}{c} \lambda_{1},\lambda_{2},\cdots ,\lambda_{n}=1\\ \left(\lambda_{1}+\lambda_{2}+\cdots +\lambda_{n}=\lambda \right) \end{array}}^{\lambda }\left(\begin{array}{c} \lambda \\ \lambda_{1},\lambda_{2},\cdots ,\lambda_{n} \end{array}\right)\cdot m_{1}^{\lambda_{1}}\cdot m_{2}^{\lambda_{2}}\cdot \ldots \cdot m_{n}^{\lambda_{n}}$$

“The probability of each such complexion is thus exactly proportional to the coefficient of the corresponding power series term, when you first form the product

(76) $$\left({m^{\prime }}_{1}+{m^{\prime }}_{2}+\ldots +{m^{\prime }}_{n}\right)\left({m^{\prime \prime }}_{1}+{m^{\prime \prime }}_{2}+\ldots +{m^{\prime \prime }}_{n}\right)\ldots \left(m_{1}^{\left(\lambda \right)}+m_{2}^{\left(\lambda \right)}+\ldots +m_{n}^{\left(\lambda \right)}\right)\;(B-A+1)$$

and finally omit from this product the upper indexes, which then generates a term exactly proportional to the polynomial coefficient.” (p. 2002f).

The polynomial coefficient is defined as

(77) $$\left(\begin{array}{c} \lambda \\ \lambda_{1},\lambda_{2},\cdots ,\lambda_{n} \end{array}\right):=\frac{\lambda !}{\lambda_{0}!\lambda_{1}!\lambda_{2}!\cdots \lambda_{n}!}$$

Notice, that these coefficients do not account for the sequence in which of the *J* draws of the balls have been drawn. Boltzmann wishes to include that and considers all of the *n^λ^* terms of the expanded product (76). He continues,

“Then by the symbol $m_{1}^{\prime }\cdot m_{3}^{\prime \prime }\cdot m_{7}^{\prime \prime \prime }\cdots$ we understand that the first pick corresponded to the first molecule, the second pick corresponded to the third molecule, on the third pick the ball corresponding to the seventh molecule was picked out, etc. All possible products of the variables symbol $m_{1}^{\prime },m_{1}^{\prime \prime },m_{2}^{\prime }$ etc. represent equi-probable complexions. We want to know how often among all the terms of the power series (A) [(74)] (whose total number is *n^λ^*), there occur terms whose coefficients contain any one state distribution. For example, consider the state distribution where one molecule has all the kinetic energy, all others have zero kinetic energy. This state distribution appears to correspond to the following members of the power series (A) [(74)]

(78) $$m_{1}^{\lambda }\cdot m_{2}^{0}\cdot m_{3}^{0}\ldots ,m_{1}^{0}\cdot m_{2}^{\lambda }\cdot m_{3}^{0}\ldots ,m_{2}^{0}\cdot m_{2}^{0}\cdot m_{3}^{\lambda }\ldots etc.\;(B-A+2)$$

with ’undivided’ *λ* [a correct translation is ‘*λ* terms in total’]. Similarly, for the state distribution in which $w_{0}$ molecules have kinetic energy zero, $w_{1}$ molecules have kinetic energy *ε*, $w_{2}$ molecules have kinetic energy 2*ε*, etc. there are

(79) $$\frac{\lambda !}{w_{0}!w_{1}!w_{2}!\cdots w_{\lambda }!}\;(B-A+3)$$

members of the power series (A).” (p. 2003). This, however, leads to the meaningless result (B-A+5)/(81). The meaningful formula (82) is obtained when using the probability (B-3)/(42) instead. 

“Each of these elements has the same polynomial coefficient, and that is identical to

(80) $$\frac{\lambda !}{{\left(0!\right)}^{w_{0}}{\left(1!\right)}^{w_{1}}{\left(2!\right)}^{w_{2}}\ldots {\left(\lambda !\right)}^{w_{\lambda }}}."\;(B-A+4)$$

(p. 2003). Usually, “polynomial coefficient” is synonymous with “multinomial coefficient”, and that is, for instance, (79). *λ*! equals the number of permutations of *λ* that are distinguished, non-interchangeable elements. ${\left(s!\right)}^{w_{s}}$ means that there are $w_{s}\times s$ elements of them not being distinguished, i.e., being interchangeable.

“In summary, therefore, according to the now accepted definition, the probability of this state distribution is

(81) $$\frac{{\left(\lambda !\right)}^{2}}{n^{\lambda }}\cdot \frac{1}{w_{0}!w_{1}!w_{2}!\ldots w_{\lambda }!}\cdot \frac{1}{{\left(0!\right)}^{w_{0}}{\left(1!\right)}^{w_{1}}{\left(2!\right)}^{w_{2}}\ldots {\left(\lambda !\right)}^{w_{\lambda }}}\;(B-A+5)$$

However, the maximization of this quantity also does not lead to the state distribution corresponding to thermal equilibrium.” (p. 2003).

Expression (B-A+5)/(81) is the product of the inverse of the number of terms in the power series (A), *n^λ^*, and expressions (B-A+3)/(79) and (B-A+4)/(80). If one uses the probability (B-3)/(42) instead of the expression (B-A+3)/(79), one obtains the probability

(82) $$P_{\mathrm{MB}}(w)=\left(\begin{array}{c} n\\ w_{0}\ldots w_{\lambda } \end{array}\right)\cdot \frac{\lambda !}{{\left(0!\right)}^{w_{0}}\cdots {\left(\lambda !\right)}^{w_{\lambda }}}\cdot {\left(\frac{1}{n}\right)}^{\lambda }$$

This is Bach’s [4] formula (3.70)/(5.9) for the probability of the occupancy number, **w**, within Maxwell–Boltzmann statistics.

### 4.7. P Yields an Extensive Entropy

Boltzmann considered the entropy of only the continuum case. For this, I refer to Planck’s lectures ‘Theory of Heat Radiation’^19^ to show that formula (42) yields an *extensive* entropy. 

For large values of $w_{s}$ and *n*, Stirling’s formula allows for simplifying formula (42) as follows:

(83) $$P={\left(\frac{n}{w_{0}}\right)}^{w_{0}}{\left(\frac{n}{w_{1}}\right)}^{w_{1}}{\left(\frac{n}{w_{2}}\right)}^{w_{2}}\ldots {\left(\frac{n}{w_{p}}\right)}^{w_{p}}\;(1991-172)$$

This form immediately yields the corresponding entropy as

(84) $$S_{P}=k_{B}\ln P=k_{B}\sum_{s=0}^{p}w_{s}\ln\left(\frac{n}{w_{s}}\right)\equiv -k_{B}n\sum_{s=0}^{p}{\bar{w}}_{s}\ln({\bar{w}}_{s});{\bar{w}}_{s}:=\frac{w_{s}}{n}\;(1991-173)$$

This is proportional to the number of molecules, *n*, since all ${\bar{w}}_{s}$ are intensive quantities.

This result is, methodologically, most important, because it is often claimed that the extensivity of the entropy of a classical gas of *n* particles needs to take a factor of 1/n! from the indistinguishability of quantum particles, as discussed at the beginning of this section. Boltzmann’s results show that this is not the case. This fact is important for the self-consistency of classical statistical mechanics which has been stressed in [29].

### 4.8. Planckian Mean Energy of a Molecule

Let us insert Formula (48) for $w_{s}$ into the entropy Equation (84) and exploit constraints (40) and (41) (cf. [24], p. 116f). This leads to the expression

(85) $$S_{P}=k_{B}\left[\left(n+\lambda \right)\ln\left(n+\lambda \right)-\lambda \ln\lambda -n\ln n\right]=k_{B}n\left[\left(1+\frac{\lambda }{n}\right)\ln\left(1+\frac{\lambda }{n}\right)-\frac{\lambda }{n}\ln\left(\frac{\lambda }{n}\right)\right]$$

With n→N and λ/n→U/ε, this is Planck’s 1901 entropy (1901-5+1)/(120) of *N* oscillators of equal frequency.

Now apply the fundamental Formula (89) in the following form:

(86) $$\frac{1}{T}={\left(\frac{dS_{P}}{dE}\right)}_{n}={\left(\frac{dS_{P}}{d\lambda }\right)}_{n}\frac{d\lambda }{dE}={\left(\frac{dS_{P}}{d\lambda }\right)}_{n}\frac{1}{\varepsilon }$$

to obtain a Planckian result for the mean kinetic energy of a molecule, *U* (cf. Planck’s 1900 implicit formula (114) for the mean radiation energy of a resonator):

(87) $$\frac{\varepsilon }{k_{B}T}=\ln\left(1+\frac{n}{\lambda }\right);U:=\frac{\lambda \varepsilon }{n}=\frac{\varepsilon }{e^{\frac{\varepsilon }{k_{B}T}}-1}$$

Of course, Boltzmann’s understanding of the physical meaning of the energy portion, *ε*, was far from Planck’s understanding.

Boltzmann preferred arguments based on a discrete view of physical quantities, cf. [16] (p. 50). “It goes without saying that these formulas are not derived here solely for finite *p* and *n* values, because these are unlikely to be of any practical importance, but rather to obtain formulas which provide the correct limiting values when *p* and *n* become infinite.” (p. 1983). As a matter of fact, in contrast to Planck’s situation in 1900, there was no experimental indication for Boltzmann to keep *ε* and also *p* finite. (See also [3].)

Thus, in his 1901 article [6], where he considers the set of resonators of a given frequency, *ν*, to build a closed thermodynamical system, Planck could have arrived at his radiation law *without* setting up a different combinatorial model, as Klein [30] (p. 473f) states without proof. As he submitted his manuscript [6] only a few weeks after his December 1900 talk [1], he was perhaps still too close to the model presented there. In it, the radiation in the resonators of *all* frequencies and the radiation in the medium surrounding them build a closed thermodynamical system. Then, it is first necessary to combine all of the subsystems in Lorentz’s manner [27] (pp. 252–254). This, however, would require assuming the radiation in the medium consists of energy elements, too. The author will return to this issue in Section 6.

## 5. Planck’s Thermodynamic Derivation of His Radiation Formula

“… wie alle Functionen es sind [von einfacher Form], die nicht von den Eigenschaften einzelner Körper abhängen, und die man bisher kennengelernt hat”. [31] (p. 292).

Planck obtained his radiation formula through extensive explorations of the thermodynamic properties of electromagnetic radiation. They provided him with relationships between various quantities that are necessary for the statistical treatment, too. Nevertheless, there is some discrepancy in the literature regarding the way in which he reached his radiation formula. This section contains a side-step to consider this.

Planck was a leading researcher on thermodynamics and, during the second half of the 1890s, pioneered the thermodynamics of electromagnetic radiation. He was rather reserved regarding statistical mechanics, although he was an atomist. This led him to a heuristic derivation of his radiation formula, which is being sketched in this section.

### 5.1. Thermodynamics of Electromagnetic Radiation

In terms of the internal energy, *U*, the fundamental thermodynamic relation reads

(88) $$dU=TdS-pdV+\sum_{i=1}^{k}\mu_{i}dN_{i}$$

where *T* indicates temperature; *S*, entropy; *p*, pressure; *V*, volume; $\mu_{i}$ and *N_i_* the chemical potential and the particle number of species *i*, respectively. In case of constant volume and particle numbers, or vanishing chemical potentials, it implies the following:

(89) $$T={\left(\frac{dU}{dS}\right)}_{V,N_{i}},\frac{1}{T}={\left(\frac{dS}{dU}\right)}_{V,N_{i}}$$

For a case in which the internal energy deviates from its equilibrium value, $U_{0}$, by only a small value, Δ*U*, we have

(90) $$U=U_{0}+\Delta U\;(1900a-7)$$

Now, Planck [32] (§ 4) approximates as follows:

(91) $$\frac{dS}{dU}={\left(\frac{dS}{dU}\right)}_{0}+{\left(\frac{d^{2}S}{dU^{2}}\right)}_{0}\Delta U$$

For the rather exotic^20^

(92) $${\left(\frac{d^{2}S}{dU^{2}}\right)}_{0}=\frac{\nu^{2}}{c^{2}}{\left(\frac{d^{2}L}{dK^{2}}\right)}_{0},\;(1900a-11)$$

where *ν* indicates radiation frequency; *c*, speed of light in vacuo; L, intensity of the radiation with frequency, *ν*, in an arbitrary direction and at an arbitrary time; K, intensity of the radiation per polarization direction. It “has got a simple physical meaning.” [35] (p. 203, fn. 2).

### 5.2. Planck’s Radiation Formula I

Planck [35] (p. 203) claims to have explored various expressions of $d^{2}S/dU^{2}$. Wien’s 1893 displacement law [36] was well established in 1900. It poses a most simple condition on the distribution law:

(93) $$\lambda_{\mathrm{peak}}T=const,$$

where $\lambda_{\mathrm{peak}}$ indicates the maximum of energy distribution over the radiation wavelength, *λ*. For this, Planck restricted his explorations of expressions for $d^{2}S/dU^{2}$ which are compatible with Wien’s law.

**Expression 1:** The formula

(94) $$\frac{d^{2}S}{dU^{2}}=-\frac{const}{U}\;(1900b-3)$$

leads to Wien’s 1896 radiation formula^21^ [46]

(95) $$K=b\frac{v^{3}}{c^{2}}e^{-a\frac{\nu }{T}};a,b=const.$$

It agrees with the then available experimental data for *small* wavelengths.

**Expression 2:** The formula

(96) $$\frac{d^{2}S}{dU^{2}}=-\frac{const}{U^{2}}$$

is compatible with Rayleigh’s 1900 [37] heuristic formula,

(97) $$K=c_{1}\frac{T}{\lambda^{4}}\exp\left\{-\frac{c_{2}}{\lambda T}\right\};c_{1,2}=const,$$

for long wavelengths.^22^

Thus, the experimental results of that time agree with the formula

(98) $$R:={\left(\frac{d^{2}S}{dU^{2}}\right)}^{-1}=-c_{1}\cdot U,c_{1}=const>0$$

at high radiation frequencies and with the formula

(99) $$R={\left(\frac{d^{2}S}{dU^{2}}\right)}^{-1}=-c_{2}\cdot U^{2},c_{2}=const>0$$

at low radiation frequencies. It is thus tempting to interpolate between both cases^23^:

(100) $$R={\left(\frac{d^{2}S}{dU^{2}}\right)}^{-1}=-aU-bU^{2}$$

or

(101) $$\frac{d^{2}S}{dU^{2}}=-\frac{\alpha }{U\left(\beta +U\right)}\;(1900b-4)$$

Using, (i), the relation

(102) $$\frac{dS}{dU}=\frac{1}{T},\;(1900b-5)$$

(ii), Wien’s displacement law in “its most general form, $S=f\left(U/\nu \right)$” [35] (p. 206, fn. 1), and, (iii), formula (1900b-4)/(101), Planck obtained “the two-parametric radiation formula”

(103) $$E=\frac{C\lambda^{-5}}{e^{c/\lambda T}-1}\;(1900b-6)$$

The “−1” in the denominator makes the crucial difference to the distribution formulae suggested by Rayleigh and Wien.

## 6. Planck’s State Definitions and Counting

“In the morning of the next day, my colleague Rubens came to see me and told me that after the end of the meeting he had compared my formula exactly with his measurement data that very night and had found a satisfactory agreement everywhere.” [40] (p. 157).^24^ His formula fitted the experimental data obtained by Rubens and Kurlbaum [44] (Fig. 2; cf. also [45]) for *all* of the wavelengths available to them, and this better than the formulae suggested by Wien 1896 [46], Thiesen 1900 [47], and Lord Rayleigh 1900 [37]. This brought Planck “some weeks of the most strenuous work of my life” [48] to find a physical justification for that formula. Having not found any other way (although being atomist, he worked solely on continuum theories), he [1] eventually resorted to Boltzmann’s 1877 [2] probabilistic approach which has been described in Section 4. He was not aware that Boltzmann’s 1868 probabilistic model [5] was much closer to his goal. In contrast to Boltzmann, he ascribed to the energy portion, also known as the energy element, a concrete physical meaning, viz., ε=hν. As a consequence, the energy element, *ε*, is *finite*, because *h* is a combination of finite empirical spectroscopical parameters.

In his December 1900 talk [1], Planck enthusiastically introduced *h* as a “natural constant” at the very beginning. Unfortunately, in his subsequent 1901 article [6], *h* was set back to § 10, where it was merely a constant of proportionality.

As a consequence, *h* has not been used in Einstein’s pioneering articles on the photo effect (1905) [49] and the specific heat of crystals (1907) [50]. In order to save classical physics, Planck tried to restrict quantum processes to the absorption and emission of electromagnetic radiation, respectively, and still rejected Einstein’s 1905 “light quantum hypothesis” in his 1913 recommendation letter for the election of Einstein to the Prussian Academy of Science [51]. The term ‘photon’ was coined later [52]. Notice that the existence of a natural constant of dimension ‘action’ emerges already from the Stefan–Boltzmann constant [53] (see also [54], p. 6 I, fn. 3). Admittedly, it is not necessary to introduce *h* at this stage, see Section 6.2.

Moreover, there are further issues in Planck’s 1900/1901 work that still deserve closer exploration as well as clarification. For the reader’s convenience, Planck’s argument will be quoted to some extent.

### 6.1. Planck’s 1900 Probabilistic Approach

“… danach zeigen Resonatoren von großer Schwingungszahl eine besondere Habgier nach Energie (wobei es ihnen dann beim Austausch der Energieelemente geschehen kann, dass sie besonders wenige davon abbekommen)”.^25^

Thus, Planck considers “a large number of monochromatically vibrating—*N* of frequency *ν*, *N*′ of frequency *ν’*, *N*″ of frequency *ν*″, …, with all *N* large number—which are at large distances apart and are enclosed in a diathermic medium with light velocity *c* and bounded by reflecting walls.^26^ Let the system contain a certain amount of energy, the total energy $E_{t}$ (erg) which is present partly in the medium as traveling radiation and partly in the resonators as vibrational energy. The question is how in a stationary state this energy is distributed over the vibrations of the resonator and the various of the radiation present in the medium, and what will be the temperature of the total system. 

To answer this question, we, first of all, consider the vibrations of the resonators and assign to them arbitrary definite energies, for instance, an energy *E* to the *N* resonators *ν*, *E*′ to the *N*′ resonators *ν*′…. The sum

(104) $$E+E^{\prime }+E^{\prime \prime }+\cdots =E_{0}\;(1900c-1)$$

must, of course, be less than $E_{t}$. The remainder $E_{t}-E_{0}$ pertains then to the radiation present in the medium. We must now give the distribution of the energy over the separate resonators of each group, first of all, the distribution of the energy *E* over the *N* resonators of frequency *ν*. If *E* [is] considered to be a continuously divisible quantity, this distribution is possible in infinitely many ways. We consider, however—this is the most essential point of the whole calculation—*E* to be composed of a very definite number of equal parts and use thereto the constant of nature *h* = 6.55 × 10^−27^ erg · sec. This constant multiplied by the common frequency *ν* of the resonators gives us the energy element *ε* in erg, and [by] dividing *E* by *ε* we get the number *P* of energy elements which must be divided over the *N* resonators.” [1] (p. 239f, En. p. 83f).

Theoretically, Planck could have applied Boltzmann’s 1868 [5] combinatorial analysis and performed the calculations in Section 3.5 separately for each set of resonators of frequency, *ν* (cf. Planck 1901 [6], § 5). However, it is by no means granted that Boltzmann’s argument applies to *non*-ponderable matter, too. For this, Planck’s work also represents an important original contribution to statistical physics [24] (p. 108).

By the way, many analyses refer to late reminiscences by Planck about his path to his radiation law. In view of his own years long fight against the physical consequences of his light quantum hypothesis, I do only partly second that. I consider Einstein’s 1905 formulation “Planck’s light quantum hypothesis” [49] not only to be a trick to evoke the authority of Planck for promoting his own light quantum hypothesis. Black-body radiation is a phenomenon that is independent of the properties of specific bodies. Its properties are thus of general nature. If, in Planck’s model, the medium would contain radiation not consisting of energy elements, *hν*, the energy elements would decay when leaving a resonator and be composed when entering it. 

Some authors interchange “resonators” and “oscillators”. The quantization of the phase space of harmonic oscillators has brought Planck to the zero-point energy. On the other hand, oscillators have additional degrees of freedom, the entropy of which needs to be accounted for as well [1].

Planck continues, “It is clear that the distribution of *P* energy elements over *N* resonators can only take place in a finite, well–defined number of ways. Each of these ways of distribution we call a “complexion”, using an expression introduced by Mr. Boltzmann for a similar quantity. If we denote the resonators by the numbers 1, 2, 3, …, *N*, and write these in a row, and if we under each resonator put the number of its energy elements, we get for each complexion a symbol of the following form.” [1] (p. 240, En. p. 84; N=10, P=100).


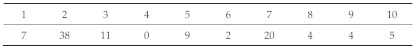


The second row represents the occupation number vector, **k**, in the combinatorial scheme. *P* “energy elements” (particles) in *N* resonators (cells), cf. Section 2.2. The constraint

(105) $$\sum_{r=1}^{N}k_{r}=P$$

corresponds to the Level 2 constraint (6) for occupation numbers.

Hence, being an occupation number vector, Planck’s complexion is *exactly the same* as Boltzmann’s 1877 [2] complexion. The actual differences in their treatments are summarized in Table 3.

Two complexions are considered to be different if the second rows contain the same numbers but in a different order. This means that the energy elements are interchangeable, while the resonators are not. In agreement with Formula (7), the number of different complexions (occupation number vectors) for this set of resonators equals the following (when *N* >> 1 and *P* >> 1):

(106) $$\left[ℜ=\right]\frac{N\cdot \left(N+1\right)\cdot \left(N+2\right)\ldots \left(N+P-1\right)}{1\cdot 2\cdot 3\ldots P}=\frac{\left(N+P-1\right)!}{\left(N-1\right)!P!}\approx \frac{{\left(N+P\right)}^{N+P}}{N^{N}P^{P}}\;(1900c-2,3)$$

This exactly corresponds with Boltzmann’s 1877 quantity, *J*, (B-15+3)/(60), where $\lambda \hat{=}P$ and $n\hat{=}N$.

The algebraic (!) symmetry of this formula in N−1 and *P* was perhaps the reason for Reiche^27^ to see no essential difference between the distribution of energy elements onto resonators, or, *vice versa*, that of resonators onto energy intervals. Since the latter are continuous, this seeming ambiguity has been stressed in discussions on the extent to which Planck’s treatment implies discontinuity, see [56] (p. 6) and also [57] (p. 243ff). However, the constraint (105) breaks that symmetry (see also the remarks on this issue in Section 2.2).

Ehrenfest writes that, “Planck gives two different derivations for his radiation formula: determination of the most probable distribution,

(i) of the resonators onto the different energy ranges,
(ii) of the energy onto the different resonators (§ 150 and § 148 in Planck’s 1906 lectures, respectively).

… The method (ii) for deriving Planck’s radiation equation is, however, completely identical to method (i): its combinatorial apparatus differs from that of method (i) only in a different way of combining the complexions to be counted.” [58] (p. 113). Unfortunately, Ehrenfest provides no details. Actually, in § 148, Planck begins with the number of resonators that have a certain amount of energy (Level 3) but continues with the remark that it is simpler to work with the distribution of the energy elements onto the resonators (Level 2), as in 1900/1901 [1,6]. In § 150, Planck partitions the phase space of a linear harmonic oscillator into elliptic rings, each of area *h*, and seeks the probability for the energy of the oscillator to lie between U=pε and U+ΔU=pε+ε. He does not calculate that probability, but states that this leads to ε=hν without using Wien’s displacement law.

One reason for Planck’s usage of occupation numbers may be the following: Integrating formula (1900b-4)/(101) twice [24] (p. 112f), or using his empirical distribution (1900b-6)/(103) (cf. [30], p. 469, (9), and p. 474), the entropy of a single resonator becomes

(107) $$S=\frac{\alpha }{\beta }\left[\left(\beta +U\right)\ln\left(\beta +U\right)-U\ln U\right]=\alpha \left[\left(1+\frac{U}{\beta }\right)\ln\left(1+\frac{U}{\beta }\right)-\frac{U}{\beta }\ln\frac{U}{\beta }\right]$$

(cf. formula (1901-6)/(121)). Accordingly, the entropy of *N* resonators equals

(108) $$S_{N}=NS=\alpha \left[\left(N+P\right)\ln\left(N+P\right)-P\ln P-N\ln N\right];P:=\frac{NU}{\beta }$$

Using Stirling’s formula, this can be written as

(109) $$S_{N}=\alpha \ln\frac{{\left(N+P\right)}^{N+P}}{N^{N}P^{P}}\approx \alpha \ln\frac{\left(N+P\right)!}{N!P!}=\alpha \ln\left(\begin{array}{c} N+P\\ N \end{array}\right)$$

Now, $\left(\begin{array}{c} N+P\\ N \end{array}\right)$ is the number of possibilities to distribute *P* interchangeable particles (energy elements) onto *N*+1 non-interchangeable cells (resonators), as shown in Section 2.2. For N≫1, the difference between *N* and *N*+1 is negligible.^28^ This argument, however, may be questionable, because Planck first follows Boltzmann’s 1877 [2] line to maximize probability and entropy, while there is nothing to vary in the entropy (109) to maximize it.

Planck considers the *whole* system “black body”, which consists of the following:

i. the radiation in all resonators,
ii. of all frequencies, *and*
iii. the radiation in the medium surrounding the resonators; it comprises *all* frequencies.

He discards the radiation in the medium in his combinatorial calculations. It is calculated from that in the resonators using formula (1900c-4)/(111). The total relative probability is the product of the relative probabilities of all resonators of all frequencies only, as follows:

(110) $${ℜ}_{0}=ℜ\cdot {ℜ}^{\prime }\cdot {ℜ}^{\prime \prime }\cdot \ldots$$

“Among all energy distributions which are possible for a constant $E_{0}=E+E^{\prime }+E^{\prime \prime }+\cdots$ there is one well-defined one for which the number of possible complexions ℜ_0_ is larger than for any other distribution. We look for this distribution, if necessary, by trial, since this will just be the distribution taken up by the resonators in the stationary radiation field if they together possess the energy $E_{0}$. This quantities *E*, *E*′, *E*″, … can then be expressed in terms of $E_{0}$. Dividing *E* by *N*, *E*′ by *N*′, … we obtain the stationary value of the energy $U_{\nu },U_{\nu \prime }^{’},U_{\nu \prime \prime }^{\prime \prime },\cdots$ of a single resonator of each group, and thus also the spatial density of the corresponding radiation energy in a diathermic medium in the spectral range *ν* to ν+dν,

(111) $$u_{\nu }d\nu =\frac{8\pi \nu^{2}}{c^{3}}\cdot U_{\nu }d\nu ,\;(1900c-4)$$

so that the energy of the medium is also determined.” (p. 241, En. p. 85).

Planck claims that all energies, *E*, *E*′, … can be calculated from $E_{0}$ (1900c-1)/(104). $E_{0}$ is found by trial and error. It is that value, for which ℜ_0_ assumes its maximum *and* the total energy equals the prescribed value, $E_{t}$. I find it simpler to calculate $E_{0}$ from ℜ_0_, to add the energy of the radiation in the medium, $E_{\mathrm{medium}}$, according to formula (1900c-6)/(113) and to assign the distribution corresponding to ℜ_0_ to the distribution for $E_{t}=E_{0}+E_{\mathrm{medium}}$.

Then Planck invokes Formula (102) in the following form (he uses ϑ for *T*; $k\equiv k_{B}$):

(112) $$\frac{1}{T}=k\frac{d\ln{ℜ}_{0}}{dE_{0}}\;\cdot (1900c-5)$$

where “… $k\ln{ℜ}_{0}$ is the entropy of the systems of resonators; it is the sum of the entropy of all separate resonators.” (p. 241, En. 85). 

The calculations above are extremely complicated, if possible, at all. “A more general calculation which is performed very simply, using the above prescription shows much more directly that the normal energy distribution determined this way for a medium containing radiation is given by the expression

(113) $$u_{\nu }d\nu =\frac{8\pi h\nu^{3}}{c^{3}}\frac{d\nu }{e^{\frac{h\nu }{k_{B}T}}-1}\;(1900c-6)$$

which corresponds exactly to the spectral formula which I give earlier…” (p. 242f, En. p. 86f.), see the Formula (103). The missing of intermediate steps of argument is obvious.

When comparing formulae (1900c-4)/(111) and (1900c-6)/(113), Planck has obtained for the average energy of a single oscillator of frequency, *ν*, the following formula:

(114) $$U_{\nu }=\frac{h\nu }{e^{\frac{h\nu }{k_{B}T}}-1}$$

I assume that Planck never calculated ℜ_0_ and $E_{0}$ and inserted them into Formula (112), but exploited the crucial simplifications he published a few weeks after his talk, as shown in the next Section.

### 6.2. Planck’s 1901 Modifications

In his 1901 article [6] (which reached the editorial office as early as 9 January), Planck presents a major modification to his December 1900 talk [1]. I will concentrate on the probabilistic aspects.

“The constant energy *U* of a single stationary vibrating resonator accordingly is to be taken as time average, or what is the same thing, as a simultaneous average of the energies of a large number *N* of identical resonators, situated in the same stationary radiation field, and which are sufficiently separated so as not to influence each other directly. It is in this sense that we shall refer to the average energy *U* of a single resonator. Then to the total energy

(115) $$U_{N}=NU\;\cdot (1901-1)$$

of such a system of *N* resonators there corresponds a certain total entropy

(116) $$S_{N}=NS\;\cdot (1901-2)$$

of the same system, where *S* represents the average entropy of a single resonator and the entropy, $S_{N}$, depends on the disorder with which the total energy $U_{N}$ is distributed among the individual resonators.” (§ 1). Thus, Planck considers the set of all resonators of *one* frequency to build a closed thermodynamical system.^29^ Now it is possible to apply Boltzmann’s 1868 [5] combinatorial calculations (see Section 3.5); perhaps, he did not know them.

“§ 2. We now set the entropy *S_N_* of the system proportional to the logarithm of its probability *W*, within an arbitrary additive constant, so that the *N* resonators together have the energy $U_{N}$ (“$E_{N}$” in the translation cited is a typo):

(117) $$S_{N}=k_{B}\ln W+\mathrm{constant}\;(1901-3)$$

In my opinion this actually serves as a definition of the probability *W*, since in the basic assumptions of electromagnetic theory there is no definite evidence for such a probability. The suitability of this expression is evident from the outset, in view of its simplicity and close connection with a theorem from kinetic gas theory.^30^

§ 3. It is now a matter of finding the probability *W* so that the *N* resonators together possess the vibrational energy $U_{N}$. Moreover, it is necessary to interpret $U_{N}$ not as a continuous, infinitely divisible quantity, but as a discrete quantity composed of an integral number of finite equal parts. Let us call each such part the energy element *ε*; consequently, we must set

(118) $$U_{N}=P\varepsilon \;(1901-4)$$

where *P* represents a large integer generally, while the value of *ε* is yet uncertain.”

Indeed, it is not yet necessary to set ε=hν, as ε~ν follows from Wien’s displacement law, as shown below.

Then, Planck reproduces his 1900 [1] probabilistic example of N=10 resonators and P=100 energy elements, see Section 6.1.

§ 4. Planck discusses the assumption that all complexions occur with the same probability.

“But should experience finally decide in favor it will be possible to draw further conclusions from the validity of this hypothesis about the particular nature of the resonator vibrations; namely in the interpretation put forth by J. v. Kries [60] regarding the character of the “original amplitudes, comparable in amplitude but independent of each other.^31^

§ 5. Then, the probability, *W*, in formula (1901-3)/(117) is proportional to the number, R, of complexions (1900c-2,3)/(106). Planck obtains “after suitable determination of the additive constant:

(119) $$S_{N}=k_{B}\ln ℜ=k_{B}\left\{\left(N+P\right)\ln\left(N+P\right)-N\ln N-P\ln P\right\}\;(1901-5)$$

and by considering (4) [(118)] and (1) [(115)}:

(120) $$S_{N}=k_{B}N\left\{\left(1+\frac{U}{\varepsilon }\right)\ln\left(1+\frac{U}{\varepsilon }\right)-\frac{U}{\varepsilon }\ln\frac{U}{\varepsilon }\right\}\;(1901-5+1)$$

Thus, according to Equation (2) [(116)] the entropy *S* of a resonator as a function of its energy *U* is given by:

(121) $$S=k_{B}\left\{\left(1+\frac{U}{\varepsilon }\right)\ln\left(1+\frac{U}{\varepsilon }\right)-\frac{U}{\varepsilon }\ln\frac{U}{\varepsilon }\right\}”\;(1901-6).$$

In his December 1900 talk, Planck followed Boltzmann’s 1877 non-equilibrium approach and tried to maximize the probability (110), as shown in Section 6.1. Now he has in mind the thermodynamic equilibrium, as Boltzmann did in his 1868 memoir, as discussed, in particular, in Section 3.5.^32^

§§ 7–9. Planck discusses Wien’s displacement law and gives him “the simplest form … known to me”,

(122) $$S=f\left(\frac{U}{\nu }\right)\;(1901-10)$$

Formula (1901-6)/(121) is compatible with this form, iff

(123) $$\varepsilon ∼\nu ,\begin{array}{ccc} & \mathrm{say}, & \end{array}\varepsilon =h\nu ;h=const$$

Inserting this into formula (1901-6)/(121) and using formula (1900b-5)/(102) yields $U_{\nu }$ (114) and, thus, Planck’s radiation formulas (1900c-6)/(113) and (1900b-6)/(103), respectively.^33^

In the last few paragraphs, Planck shows that his formulas yield numerical values for the natural constants involved that are in good agreement with the experimental data. In fact, they are the most accurate theoretical values of that time.

## 7. Summary and Conclusions

It seems that Truesdell’s thoughts on Newton’s ‘Principia’ can also be said about Boltzmann’s and Planck’s articles. “Since the Principia is one of those works everyone talks of but no one reads, anything said about it other than the usual honey-sauced eulogy must stand up against righteous indignation from all sides. But it is a work of science, not a bible. It should be studied and weighed—admired, indeed, but not sworn upon. It has its novelties and its repetitions, its elegant perfections and its errors, its lightning abbreviations and its needless detours, its extraordinary standards of rigor and its logical gaps, its elimination of stated hypotheses and its introduction of unstated ones.” [61].

Thus, why did Planck succeded in finding his distribution law using probabilistic methods, while Boltzmann, to whom he refers, did not obtain it? Did they exploit different probabilistic schemes, or is it merely a consequence of keeping the energy elements finite? To uncover this, I have analyzed some of the most relevant contributions of Boltzmann and Planck to the manner of state definition and counting. Which manner is the correct one?

Already Boltzmann’s pioneering 1868 memoir [5], written at age of 24, contains eminent results. “To demonstrate the compatibility of the assumption of a microscopic atomistic structure (he was a passionate proponent of atomism), Boltzmann derives in 1868 … the Maxwell velocity distribution in two dimensions … from a discrete setting. … Assuming all distributions of the *n* identical energy elements onto the *d* molecules to be *a priori* equal probable, he evaluates the cardinality of the set of sequences of occupation numbers and obtains the probability distribution^34^

(124) $$P_{P}(K=k)={\left(\begin{array}{c} d+n-1\\ n \end{array}\right)}^{-1}.\;(1997-5.2)$$

(**K** being the occupation number random variable). Second, he determines the marginal distribution of the number of energy elements in an arbitrarily chosen cell … [4] (p. 133).

(125) $$P_{P}(K=k)={\left(\begin{array}{c} d+n-1\\ n \end{array}\right)}^{-1}\left(\begin{array}{c} d+n-k-2\\ n-k \end{array}\right).”\;(1997-5.3)$$

This equilibrium theory already bears all the ingredients for obtaining Planck’s 1901 [6] entropy and distribution law (without ε=hν, of course).

Later, when replying to Loschmidt’s reversibility paradox, Boltzmann 1877 [2] argues that the macroscopic equilibrium state of a system can be deduced from its most probable microstate. The probability, $P_{P}\left(K=k\right)$ (1997-5.2)/(124), is independent of **k** and hence not suitable for that purpose. It is appropriate for systems in equilibrium, but not for systems in non-equilibrium. A most probable microstate can be calculated when using occupancy numbers. Boltzmann does so in his famous 1877 memoir [2], Section I. Here, “discrete symmetric probabilities became the foundation of the theory” [4] (p. 133). The fundamental probability distribution is the uniform distribution of the occupation numbers (complexions, Planck statistics). He evaluates the distribution of the occupancy numbers as

(126) $$P_{P}(Z=z)=\left(\begin{array}{c} d\\ z_{0}\cdots z_{n} \end{array}\right){\left(\begin{array}{c} d+n-1\\ n \end{array}\right)}^{-1},\;(1997-5.6)$$

where **Z** is the occupancy number random variable, cf. formula (B-3)/(42). The most probable state, z*, is that with the maximum probability, i.e., the maximum of $P_{P}$ with respect to **z**. This can lead to Planck’s distribution law for the energy elements, as shown in Section 4.8. Boltzmann, however, goes over to the continuum limit. Here, he finds the fundamental result that the combinatorial entropy, up to a factor and an additive constant, equals the entropy of phenomenological thermostatics in two dimensions. 

In Section IV, Boltzmann obtains expressions that do not correspond to equilibrium distributions. This could be due to various confusions that are common in pioneering work that goes so far ahead. As shown in Section 4.6, his argument can be corrected such that it yields Bach’s Maxwell–Boltzmann probability (82) of occupancy numbers.

“Just so in the case of today’s so-called Boltzmann statistics Boltzmann breaks off in 1877!” [41] (p. 215; quoted after [3], p. 23, fn. 123). “Concerning the part on discrete probabilities, it seems that Boltzmann had only three readers. These were Planck, Lorentz and Natanson.” [3] (p. 31). There are many more aspects of the relationship between Boltzmann’s work and quantum statistics [3,4,10,30,41,42,45] that deserve further exploration.

Planck’s 1900/1901 [1,6] complexions are the same as Boltzmann’s 1877 [2] complexions, viz., occupation number vectors (Level 2). However, in Boltzmann’s 1877 memoir [2], complexions (Level 2) describe microstates, while macrostates are represented by occupancy numbers (Level 3). In contrast, in Planck’s 1901 article [6], complexions (Level 2) describe macrostates, while microstates are represented by configuration numbers (Level 1).

The most probable reason for that difference is Planck’s empirical expression (107) for the entropy. Moreover, Planck considers the equilibrium state of black-body radiation and does not need Boltzmann’s 1877 [2] combinatorics for equilibrium and non-equilibrium states. Boltzmann’s 1868 [5] combinatorics for equilibrium states was obviously unknown to him.

As a matter of fact, any formula for the probability, *W*, which interrelates the entropy, *S*, and the distribution function, *f*, in the form

(127) $$S=k_{B}\left\{\left(1+f\right)\ln\left(1+f\right)-f\ln f\right\},$$

leads to the Planckian formula (87) for the average energy, *U*, of a cell (molecule, resonator). For instance, Debye [62], neglecting “−1”, applied Planck’s formula (1900c-2,3)/(106) in the form

(128) $$w_{\nu }=\frac{\left(N_{\nu }d\nu +N_{\nu }f_{\nu }d\nu \right)!}{\left(N_{\nu }d\nu \right)!\left(N_{\nu }f_{\nu }d\nu \right)!};N_{\nu }d\nu ≙N,N_{\nu }f_{\nu }d\nu ≙P\;(\mathrm{Debye}-3)$$

to obtain Formula (127). Together with the number of radiation modes in a cubic resonator, the “hypothesis of elementary quanta”, ε=hν, and 1/T=dS/dU, he arrived at Planck’s radiation law.

Both Boltzmann’s and Planck’s combinatorial calculations represent models, neither of which are *proof* of the correctness of the results. “It cannot be denied that there is a certain arbitrariness in this derivation; for one can arrange such a lottery game according to different principles. ...The certain assumption we have made about the lottery is therefore theoretically unjustifiable; it is a makeshift which cannot be avoided because we do not know the real processes.” [27] (p. 255). “In all of this, the goal must remain to replace probability considerations with consideration of the real processes …”^35^

Spałek claims that “only the explicit inclusion of the indistinguishability principle enlightens the difference between the original approach due to Boltzmann, defining the classical statistics, and its quantum correspondent.” [63] (p. 430). This is, at least, misleading. Using occupancy numbers, Boltzmann 1877 [2] implicitly assumed that both the particles (energy portions) and the cells (molecules) can be treated as being interchangeable. When keeping the energy portions finite, this leads to a *quantum* distribution law, as shown in Section 4.8.

Spałek’s claim points to the issue of whether classical statistical mechanics is capable of coping with the interchangeability of equal classical particles (being treated in detail by Bach [4]). If not, classical statistical mechanics is *not* self-consistent, but needs quantum arguments, notably, for resolving Gibbs’ paradox. However, this is *not* the case, as shown in Section 4.7 and [7] (p. 1974).

The issue of (in)distinguishability was risen first by Natanson in 1911 [64]. Unfortunately, he confused the (in)distinguishability of particles with that of states. As a consequence, classical (quantum) particles have been *principally* considered to be (in)distinguishable. Bach [4,21] demonstrates that this is not the case; for simpler expositions, see [29,33].

One may ask why Planck did not go the following way (cf. [27,50]). The relative probability that one resonator hosts *n* energy elements, *ε*, equals

(129) $$w_{n}=e^{-n\varepsilon /k_{B}T}$$

If *n* is continuous (ε→0), the average number of energy elements on the resonator and its average energy become

(130) $${\left.\langle n\rangle \right|}_{\varepsilon \to 0}=\frac{\int_{0}^{\infty }nw_{n}dn}{\int_{0}^{\infty }w_{n}dn}=\frac{k_{B}T}{\varepsilon };{\left.U\right|}_{\varepsilon \to 0}={\left.\langle n\varepsilon \rangle \right|}_{\varepsilon \to 0}=k_{B}T$$

This is the classic, invalid result. In contrast, if *n* is discrete (ε>0), the average number of energy elements on the resonator and its average energy assume their correct values.

(131) $${\left.\langle n\rangle \right|}_{\varepsilon >0}=\frac{\sum_{n=0}^{\infty }nw_{n}}{\sum_{n=0}^{\infty }w_{n}}=\frac{1}{e^{\frac{\varepsilon }{k_{B}T}}-1};{\left.U\right|}_{\varepsilon >0}={\left.\langle n\varepsilon \rangle \right|}_{\varepsilon >0}=\frac{\varepsilon }{e^{\frac{\varepsilon }{k_{B}T}}-1}$$

However, scientific research is not that simple. Boltzmann’s 1868 [5] and 1877 [2] as well as Planck’s 1900/1901 [1,6] contributions are pioneering works. They cut aisles through the thicket where roads are being built later.

## Figures and Tables

**Table 1:** The 15 different “state distributions” for the case n=λ=p=7. The numbers in columns 2–8 are not the occupancy numbers, $\left(w_{0},\cdots ,w_{7}\right)$, but the occupation numbers, $\left(k_{1},\cdots ,k_{7}\right)$ (Level 2). “The state distributions are so arranged that, read as a number, the rows are arranged in increasing order.”

#	k_1_	*k*_2_	*k*_3_	*k*_4_	*k*_5_	*k*_6_	*k*_7_	P
1	0	0	0	0	0	0	7	7
2	0	0	0	0	0	1	6	42
3	0	0	0	0	0	2	5	42
4	0	0	0	0	0	3	4	42
5	0	0	0	0	1	1	5	105
6	0	0	0	0	1	2	4	210
7	0	0	0	0	1	3	3	105
8	0	0	0	0	2	2	3	105
9	0	0	0	1	1	1	4	140
10	0	0	0	1	1	2	3	420
11	0	0	0	1	2	2	2	140
12	0	0	1	1	1	1	3	105
13	0	0	1	1	1	2	2	210
14	0	1	1	1	1	1	2	42
15	1	1	1	1	1	1	1	1

**Table 2:** The occupancy numbers, $\left(w_{0},\cdots ,w_{7}\right)$, for Boltzmann’s 15 “state distributions” in Table 1. The rows have been reordered according to the increasing value of P, to demonstrate the fact that occupancy number vectors, $w=\left(w_{0},\cdots ,w_{7}\right),$ which differ just in the sequence of their numbers, have the same probability, P, cf. formula (42) below. The equality of P = 210 in rows 6 and 13 is by chance.

#	*w*_0_	w_1_	*w*_2_	*w*_3_	*w*_4_	*w*_5_	*w*_6_	*w*_7_	P
15	0	7	0	0	0	0	0	0	1
1	6	0	0	0	0	0	0	1	7
2	5	1	0	0	0	0	1	0	42
3	5	0	1	0	0	1	0	0	42
4	5	0	0	1	1	0	0	0	42
14	1	5	1	0	0	0	0	0	42
5	4	2	0	0	0	1	0	0	105
7	4	1	0	2	0	0	0	0	105
8	4	0	2	1	0	0	0	0	105
12	2	4	0	1	0	0	0	0	105
9	3	3	0	0	1	0	0	0	140
11	3	1	3	0	0	0	0	0	140
6	4	1	1	0	1	0	0	0	210
13	2	3	2	0	0	0	0	0	210
10	3	2	1	1	0	0	0	0	420

**Table 3:** Complexion, microstate, and macrostate in Boltzmann [2,5] and Planck [1,6].

Planck versus Boltzmann	Complexion	Microstate	Macrostate
Boltzmann 1868	n. a.	configuration	occupation
Boltzmann 1877, Section I	occupation number	*occupation*	*occupancy*
Planck 1900/1901	occupation number	configuration	occupation

## Notes

1 Maxwell to Tait, 1873, quoted after [10], p. 83.

2 For good crash courses on combinatorics, see [11,12]. Feller’s classic book [13] is com-prehensive but the sequence of ex-amples and theorems is unconventional.

3 “1868” in the original text is a typo; there, I have seen only the occupation numbers, see Section 3. The 1877 memoir [2] will be considered in Section 4. Bach’s notation is replaced with Boltzmann’s 1877 [2] ones: n→λ,d→n. Planck [1,6] distributes P energy elements aka particles onto N resonators aka cells.

4 I’m using the notion ‘equal’ in the sense of Helmholtz, H. v. Einleitung zu den Vorlesungen über Theoretische Physik (Vorlesungen über Theoretische Physik, Vol. I/1; König, A., Runge, C., Eds.; Barth: Leipzig, 1903, pp. 1–50. Reprint in: [14], pp. 11–62. Equal classical particles are still distinguished by their location. Identical particles are—strictly speaking—equal in all their properties.

5 https://en.wikipedia.org/wiki/Stars_and_bars_(combinatorics), Theorem 2 (accessed on 3 March 2021). [13] (p. 38, eq. (5.2)). See also [15] especially w.r.t. Planck’s combinatorics.

6 For a more detailed account, see [3] (p. 13f).

7 Actually, Ehrenfest & Kamerlingh Onnes [14] do that for Planck’s result, i.e., for Q_2.

8 There is a 1984 Russian edition of collected works [20].

9 An exception is Bach [3,4,21,22].

10 All quotations refer to the translation by Sharp & Matschinsky [7]. Boltzmann’s equation numbers are indicated as (B-*). Several formulas are added for the sake of clarity of expla-nation.

11 This view is seconded in the report on this article in Fortschr. Physik in 1877, 1882, pp. 671–678.

12 This does not mean, that a complexion is represented by the occupancy numbers, *w*_0_, …, *w_p_*, but refers to the fact that the the occupancy numbers follow from the occupation numbers (complexion), see formula (13).

13 Descartes’ rule of signs asserts that if the nonzero terms of a single-variable polynomial with real coefficients are ordered by descending variable exponent, then the number of positive roots of the polynomial is either equal to the number of sign changes between consecutive (nonzero) coefficients, or is less than it by an even number. A root of multi-plicity k is counted as k roots. Boltzmann takes into account that pn−λ>0, since pn≫λ, see formula (54).

14 Sharp & Matschinsky [7] write “since”. In contrast, Boltzmann begins a new paragraph ([18], Vol. II, p. 186).

15 A more exact translation of the original word “mannigfaltig” [18] (Vol. II, p. 211) is ‘di-versely’ or ‘multifariously’.

16 The translation of the original “gleichbeschaffene Kugeln” [18] (Vol. II, p. 211) to “identical balls” may suggest the balls to be indistinguishable. This, however, would contradict the second sentence. Actually, “gleichbeschaffen” (being equal) means that the probability of being drawn is the same for all balls. Both the molecules and the balls are distinguished (e.g., by numbers); otherwise, it would not be possible that “every ball corresponds to a certain molecule”.

17 A more accurate translation is this: One of the various possible complexions will be that the ball corresponding to the first molecule has been drawn in all *λ* moves).

18 This corresponds to the occupation numbers in Section I with p=λ: $m_{1}^{\lambda }$ means $k_{1}=\lambda$, etc.

19 Planck, M. Vorlesungen über die Theorie der Wärmestrahlung. Leipzig: Barth, 1906. En: The Theory of Heat Radiation (authorized transl. by M. Masius), Blakiston 1914, reprints: Mineola, N.Y.: Dover 1959, 1991. I will refer to the 1991 Dover edition. (1991-n) denotes formula (n) therein. At once, I will stay with Boltzmann’s notations.

20 I am not aware of its use in thermodynamics before and after Planck. It has been intro-duced by Planck in a 1900 article [32] which is not contained in the collection [33] on Planck’s way to his radiation formula. There is a Russian translation by R. B. Segalya in [34], pp. 234–248) second derivative Planck finds.

21 Wien writes the distribution over the wavelength, λ: $\varphi_{\lambda }=\left(C/\lambda^{5}\right)\exp\left\{-c/\lambda T\right\}$ (*C*, *c*—constants).

22 For $\lambda \gg c_{2}/T$, it becomes the Rayleigh–Jeans formula, which, however, was pub-lished only by Jeans in 1905 [38]. Rubens informed Planck about that during his visit on 7 October 1900 (after [39], p. 1036 I, confirmed in [40], p. 156). After Kangro [41] (1970, Sect. 8.8; 1976, pp. 198–200), Planck’s student “Hettner [39] obtained the information from Planck himself.” (quoted after [42], p. 43, fn. 54).

23 I tend to agree with https://en.wikipedia.org/wiki/Planck%27s_law (accessed on 2 June 2021) that “Planck perhaps patched together these two heuristic formulas”, citing Hettner 1922 (see [39], p. 1036 II, formula (8)), and [43]. Planck’s 1943 memories [40] (p. 156) second that view.

24 Original text: “Am Morgen des nächsten Tages suchte mich der Kollege Rubens auf und erzählte, daß er nach dem Schluß der Sitzung noch in der nämlichen Nacht meine Formel mit seinen Messungsdaten genau verglichen und überall eine befriedigende Überein-stimmung gefunden habe”.

25 [27], p. 238 (5th lecture); En.: … according to this, resonators with a large number of vibra-tions show particular greed for energy (whereby it can then happen to them during the exchange of the energy elements that they get particularly few of them).

26 Notice, that—contrary to numerous representations—the resonators are not in the walls. Ehrenfest [54] (p. 3 I) writes, “Die Resonatoren innerhalb der Spiegelhülle leisten dasselbe wie eine leere Spiegelhülle, die an einzelnen Stellen diffus reflektiert.”—Engl.: The resona-tors inside the mirror shell perform the same as an empty mirror shell which reflects diffusely at single places—The cavity, however, has got infinitely many ultraviolet har-monics. (p. 6 II) This rules out that equivalence.

27 Remark 4 to Planck 1901 [6] in [55].

28 This way has perhaps first been described by Rosenfeld in 1936 [59] (p. 167), it is con-firmed by Planck 1943 [40] (Section III).

29 For some difficulties of this assumption, see [54], pp. 2 II–4 I.

30 Here, Planck (fn. 8) refers to p. 428 of Boltzmann’s 1877 memoir [2] analyzed in Section 4.

31 A correct translation is ’indifferent and, according to their size, comparable original mar-gins’ [60] (p. 36). Kries‘ original text [60] in more detail: “Als Gesamt-Ergebnis der logischen Untersuchung erhalten wir somit den Satz, dass Annahmen in einem zahlen-mäßig angebbaren Wahrscheinlichkeits-Verhältnis stehen, wenn sie indifferente und ihrer Größe nach vergleichbare ursprüngliche Spielräume umfassen, und dass bestimmte Wahrscheinlichkeits-Werte sich daher da ergeben, wo die Gesamtheit aller Möglichkeiten durch eine Anzahl solcher Annahmen ausgeschöpft werden kann.“ (p. 36) En.: As an overall result of the logical investigation, we thus obtain the proposition that assumptions are in a numerically specifiable probability ratio if they comprise indifferent and, accord-ing to their size, comparable original margins, and that certain probability values there-fore arise where the totality of all possibilities can be exhausted by a number of such as-sumptions. Later, Kries repeats, “Als Grundlage unserer Theorie ist der Satz zu betrachten, dass Annahmen, welche gleiche und indifferente ursprüngliche Spielräume umfassen, gleich wahrscheinlich sind.” (p. 157) En.: The basis of our theory is the theorem that assumptions involving equal and indifferent original margins are equally probable.—Planck correctly quotes Kries’ text.

32 Later, Planck [51] (Pt. III, Ch. IV) argues that the number of complexions at thermodynam-ic equilibrium is very much larger than the number of complexions at non-equilibrium. For this, “the number of all possible complexions is a good approximation to the number of complexions at thermodynamic equilibrium and thus to the maximum “thermody-namic probability””.

33 Ehrenfest [54] (p. 6 II) points to the fact, that ε→∞ for ν→∞ and that that does not rise difficulties, since *U_v_* (114) does not diverge.

34 I replace Bach’s “Bose-Einstein” with ‘Planck(ian)’, because there is no chemical poten-tial.

35 Original text: “Es ist nicht zu leugnen, dass in dieser Ableitung eine gewisse Willkür steckt; denn man kann ein solches Lotter-iespiel nach verschiedenen Grundsätzen ein-richten. … Die bestimmte Annahme, die wir über die Verlosung gemacht haben, ist also theoretisch nicht zu begründen; sie ist ein Notbehelf, der nicht zu umgehen ist, weil wir die wirklichen Vorgänge nicht kennen.” (p. 255) “Bei alledem muss es das Ziel bleiben, die Wahrscheinlichkeitsbetrachtungen durch die Betrachtung der realen Vorgänge zu ersetzen …” (p. 257).
